# Differential Contribution of the Parental Genomes to a *S. cerevisiae* × *S. uvarum* Hybrid, Inferred by Phenomic, Genomic, and Transcriptomic Analyses, at Different Industrial Stress Conditions

**DOI:** 10.3389/fbioe.2020.00129

**Published:** 2020-03-03

**Authors:** María Lairón-Peris, Laura Pérez-Través, Sara Muñiz-Calvo, José Manuel Guillamón, José María Heras, Eladio Barrio, Amparo Querol

**Affiliations:** ^1^Departamento de Biotecnología de Alimentos, Instituto de Agroquímica y Tecnología de Alimentos, CSIC, Valencia, Spain; ^2^Lallemand Bio, S.L., Madrid, Spain; ^3^Departament de Genètica, Universitat de València, Valencia, Spain

**Keywords:** *Saccharomyces cerevisiae*, *S. uvarum*, artificial hybrid, wine fermentation, ethanol tolerance, genome sequencing, RNA-seq

## Abstract

In European regions of cold climate, *S. uvarum* can replace *S. cerevisiae* in wine fermentations performed at low temperatures. *S. uvarum* is a cryotolerant yeast that produces more glycerol, less acetic acid and exhibits a better aroma profile. However, this species exhibits a poor ethanol tolerance compared with *S. cerevisiae*. In the present study, we obtained by rare mating (non-GMO strategy), and a subsequent sporulation, an interspecific *S. cerevisiae* × *S. uvarum* spore-derivative hybrid that improves or maintains a combination of parental traits of interest for the wine industry, such as good fermentation performance, increased ethanol tolerance, and high glycerol and aroma productions. Genomic sequencing analysis showed that the artificial spore-derivative hybrid is an allotriploid, which is very common among natural hybrids. Its genome contains one genome copy from the *S. uvarum* parental genome and two heterozygous copies of the *S. cerevisiae* parental genome, with the exception of a monosomic *S. cerevisiae* chromosome III, where the sex-determining *MAT* locus is located. This genome constitution supports that the original hybrid from which the spore was obtained likely originated by a rare-mating event between a mating-competent *S. cerevisiae* diploid cell and either a diploid or a haploid *S. uvarum* cell of the opposite mating type. Moreover, a comparative transcriptomic analysis reveals that each spore-derivative hybrid subgenome is regulating different processes during the fermentation, in which each parental species has demonstrated to be more efficient. Therefore, interactions between the two subgenomes in the spore-derivative hybrid improve those differential species-specific adaptations to the wine fermentation environments, already present in the parental species.

## Introduction

Wine fermentation is a complex process in which yeasts have the most predominant role (Cavalieri et al., 2003). Traditionally, yeasts present on grapes spontaneously convert sugars into ethanol and carbon dioxide, as well as other metabolites, such as glycerol, acetate, succinate, pyruvate, higher alcohols, and esters (Pretorius and Lambrechts, 2000). *Saccharomyces cerevisiae* is the predominant yeast in most wine fermentations (Pretorius, 2000), however, in cold areas, it is frequently replaced by *S. uvarum* (Rainieri, 1999; Origone et al., 2017), or its hybrids with *S. kudriavzevii* and *S. uvarum* (Masneuf et al., 1998; Demuyter et al., 2004; Antunovics et al., 2005; Gonzalez et al., 2007; Le Jeune et al., 2007; Lopandic et al., 2007; Sipiczki, 2008; Erny et al., 2012; Peris et al., 2012).

Wine *S. cerevisiae* and *S. uvarum* strains are adapted to grow in wine fermentation environments, characterized by high sugar contents, low pH, and high sulfur dioxide concentrations (Pérez-Torrado et al., 2015, 2018; Querol et al., 2018; Alonso-del-Real et al., 2019; Morard et al., 2019). However, each *Saccharomyces* species exhibits unique physiological properties that give the final wine different characteristics. The most important differences between these two species are ethanol tolerance and optimal growth temperature. *S. cerevisiae* exhibits a higher optimum growth temperature and higher ethanol tolerance (up to 15%) (Belloch et al., 2008; Arroyo-López et al., 2010; Salvadó et al., 2011a), which explains its dominance at high fermentation temperatures.

The present challenges in the wine industry are related to the effects of global climate change on winemaking and to consumer’s preferences. The global climate change has different effects on grapevines, which include a lower acidity, an altered phenolic maturation, a different tannin content, and notably, higher sugar levels by the time of harvest, especially in warm climates (Jones et al., 2005; Mozell and Thachn, 2014). At the same time, consumers prefer wines with less ethanol content and fruitier aromas. The excess of ethanol compromises the perception of wine aromatic complexity, as well as rejection by health-conscious consumers, road safety considerations, or trade barriers and taxes. To face these challenges, yeasts may have an important role. Thus, a new trend to respond to the wine industry demands is the selection of yeasts which reunite different characteristics, such as a lower ethanol yield, a higher glycerol production- to mask astringency due to unripe tannins- and which exhibit a more complex aromatic profile (Querol et al., 2018). However, these properties are not so frequent among wine *S. cerevisiae* strains, because they were unconsciously selected for millennia by humans to produce increasing amounts of ethanol in the warm climate regions, Fertile Crescent and Mediterranean basin, where vines were domesticated and winemaking was developed (This et al., 2006).

A possible solution to fulfill the wine industry demands comes from the use of wine *S. uvarum* strains, which exhibit interesting enological properties. *S. uvarum* is considered a cryotolerant yeast (Salvadó et al., 2011b) with several enological advantages over *S. cerevisiae*, such as lower ethanol and acetic acid productions, and higher glycerol and succinic acid synthesis (Bertolini et al., 1996). This species also produces high levels of a larger variety of fermentative volatiles, e.g. phenyl ethanol and phenylacetate (Masneuf-Pomarède et al., 2010; Gamero et al., 2013; Stribny et al., 2015). Nonetheless, the most important limitation of *S. uvarum* as a starter to conduct wine fermentation is its lower ethanol tolerance (Arroyo-López et al., 2010), which explains why it is outcompeted by *S. cerevisiae* in wine fermentations performed at temperatures > 20°C (Alonso-del-Real et al., 2017), as in the production of red wines. Therefore, an ethanol tolerance improvement in *S. uvarum* would be an important achievement for its beneficial use in the wine industries.

Ethanol tolerance is a quantitative trait determined by > 200 genes involved in many different cellular processes affected by ethanol (Snoek et al., 2016). Although many efforts have been made, mechanisms of ethanol tolerance are hardly understood yet.

Hybridization between *Saccharomyces* species has been proposed as adaptation mechanisms to different stresses (Sipiczki, 2008). As mentioned, natural hybrids between *S. cerevisiae* and *S. uvarum* or *S. kudriavzevii* are present in, and even dominate, wine fermentations at low temperatures in regions of Continental or Oceanic climates (Masneuf et al., 1998; Gonzalez et al., 2007; Le Jeune et al., 2007; Lopandic et al., 2007; Erny et al., 2012; Peris et al., 2012). The physiological and enological characterization of these hybrids showed that they inherited the ethanol tolerance and a good fermentation performance from *S. cerevisiae*, and adaptation to grow at low temperatures from *S. uvarum* and *S. kudriavzevii* (Pérez-Torrado et al., 2018; Querol et al., 2018). This observation prompted artificial hybridization as a good approach to improve industrial yeasts (Steensels et al., 2014). This way, in previous works, S. *cerevisiae* × *S. uvarum* hybrids were generated, by different methods (Sipiczki, 2008; Origone et al., 2018), to improve cryotolorance in wine *S. cerevisiae* strains (Kishimoto, 1994; Sebastiani et al., 2002; Solieri et al., 2005; Origone et al., 2018; García-Ríos et al., 2019).

In the present study, we used artificial hybridization of a commercial wine *S. uvarum* strain with a *S. cerevisiae* strain to improve its ethanol tolerance. This commercial *S. uvarum* strain, Velluto BMV58^TM^, is characterized by its low ethanol yield and high glycerol production in wines at the industrial level, improving the roundness and a soft mid-palate. It also produces richer secondary aromas, which confer floral and fruity notes to wines. Although this strain possesses all these interesting properties, which fulfill the consumers’ demands, its ethanol tolerance during wine fermentation is low. To improve its ethanol tolerance, we selected a highly alcohol-tolerant *S. cerevisiae* strain to obtain an interspecies hybrid with the properties of both parents. Hybrids were obtained by rare-mating and subsequently sporulated to obtain diverse hybrid derivatives. The rare-mating hybrids, their spore derivatives and the parental strains were physiologically characterized, and one spore-derivative hybrid, H14A7, was selected because it shows the best fermentative profile, an improved ethanol tolerance, and a higher glycerol yield. The genomes of this spore-derivative hybrid, as well as those of the parental *S. uvarum* and *S. cerevisiae* strains, were sequenced to determine which is the genome composition of the hybrid compared to its parents. Finally, we also analyzed the transcriptomic response of the spore-derivative hybrid during wine fermentations performed at two different temperatures, 15 and 25°C, to be compared with its parental strains under the same fermentation conditions.

## Materials and Methods

### Yeast Strains

The strains used in the present work were the *S. uvarum* wine strain BMV58 (Velluto BMV58^TM^ from Lallemand), a commercial wine strain that was selected in our laboratory, and three wine *S. cerevisiae* strains, AJ4, AJB, and AJW, provided by Lallemand Inc.

### Sporulation Assays

Yeast cells were incubated on acetate medium (1% sodium acetate, 0.1% Glucose, 0.125% yeast extract, and 2% agar) for 5–7 days at 25°C to induce sporulation. 16 asci were collected for each strain when they were present. Ascus wall was digested with β 1,3-glucuronidase (Sigma) adjusted to 2 mg mL-1, and spores were then dissected in GPY agar plates with a Singer MSM manual micromanipulator. Spores were incubated at 28°C for 3–5 days, and then, their viability was measured as the percentage of spores able to form colonies.

### *MAT* Locus Analysis

DNA from each strain was extracted according to Querol et al. (1992). The *MAT* locus was amplified with the same ‘MATalpha’ (5′- GCACGGAATATGGGACTACTTCG -3′) primer described for *S. cerevisiae* by Huxley et al. (1990), but with degenerated ‘MATalpha’ (5′-ACTCCRCTTCAAGAGTYTG -3′), and ‘MAT common’ primers (5′- AGTCACATCAAGATCRTTTATG -3′) to also allow the amplification of the *MAT* locus from *S. uvarum*.

PCR reactions were performed in 100 μl final volume following the NZYTAqII DNA polymerase supplier instructions, under the following conditions: initial denaturing at 94°C for 5 min, then 30 PCR cycles with the following steps: denaturing at 94°C for 30 s, annealing at 58°C for 30 s and extension at 72°C for 30 s; and a final extension at 72°C for 7 min.

The *S. cerevisiae* and *S. uvarum MAT* locus were differentiated by restriction analysis with endonuclease *Mse*I. Simple digestions of the PCR products with *Mse*I (FastDigest SaqAI, Thermo Scientific) were performed with 15μl of amplified DNA to a final volume of 20μl at 37°C according to supplier’s instructions. Restrictions fragments were separated on 3% agarose gel in 0.5× TBE buffer and a mixture of 50-bp 100-bp DNA ladder markers (Roche Molecular Biochemicals, Mannheim, Germany) served as size standards.

### Evaluation of Ethanol Tolerance

Ethanol tolerance of the strains was evaluated by performing growing tests in synthetic must (SM) with 10 g L^–1^ glucose, 20 g L^–1^fructose, and 60 mg L^–1^potassium metabisulfite, and increasing ethanol concentrations [0 to 10, 12, 15, and 20% (v/v)]. Strain growth was monitored by measuring absorbance at 600 nm in a SPECTROstar Omega instrument (BMG Labtech, Offenburg, Germany). The wells of the microplate were filled with 0.25 mL of SM and inoculated with 1 × 10^6^cells mL^–1^ for each strain and ethanol concentration. The experiments were performed at 15 and 25°C. Uninoculated wells were included in every plaque as a negative control to establish a threshold to discard OD_600_ values due to background noise. Measurements were taken every 30 min during over 3 days, after a pre-shaking of 20 s. The overall yeast growth was estimated as the area under the OD vs. time curve using Origin Pro 8.0 software (OriginLab Corp., Northampton MA), and the NIC and MIC parameters were obtained as described elsewhere (Arroyo-López et al., 2010). The most ethanol tolerant *S. cerevisiae* strain was subsequently used for the hybridization experiments.

### Hybridization by Rare-Mating

For the selection of natural auxotrophic markers, cells were grown on 15 mL of GPY medium (% w/v: 0.5 yeast extract, 0.5 peptone, 2 glucose) for 5 days at 28°C. One milliliter of each culture was seeded in 15 mL of fresh GPY medium and incubated again in the same conditions. This process was repeated 10 times. At the 5th and subsequent repetitions, aliquots of each culture were seeded onto α-aminoadipic (α-AA), 5-fluoroanthranilic acid (5-FAA) and 5-fluoroorotic acid (5-FOA) agar plates to select natural lys^–^, trp^–^, and ura^–^ mutant colonies, respectively (Zaret and Sherman, 1985; Boeke et al., 1987; Toyn et al., 2000). Colonies were grown on α-AA, 5-FAA or 5-FOA plates and picked again on a new α-AA, 5-FAA or 5-FOA plate, respectively. Auxotrophies were confirmed by spotting a cell suspension onto GPY-A (GPY medium with 2% w/v agar-agar), minimal medium (MM; 0.17% Yeast Nitrogen Base without amino acids, 2% glucose and 2% agar) and MM supplemented with proline (1 g L^–1^), and uracil (10 mg L^–1^), lysine (30 mg L^–1^) or tryptophan (30 mg L^–1^), depending on the auxotrophy. Plates were incubated for 5 days at 28°C.

Auxotrophic colonies were grown separately in 25 mL GPY broth for 48 h at 28°C. Cells were recovered by centrifugation and suspended in the residual supernatant. Pairs of yeast cultures to be hybridized were placed together in the same tube and aliquots of these mixed strains were inoculated in 2 mL of fresh GPY medium. After 5–10 days of static incubation at 28°C in a slanted position, cells were recovered by centrifugation, washed in sterile water, suspended in 1 mL of starvation medium and incubated for 2 h.

The parental strains AJ4 and BMV58 were assayed for sporulation in the rich GPY medium used for the rare-mating. In the case of AJ4, no sign of sporulation was detected after more than ten days, however, sporulation efficiency for BMV58 was very low and difficult to observe in this medium, but a few asci were present.

A concentrated suspension of the mixed culture was spread on MM plates and incubated at 28°C. Prototrophic colonies usually appeared after 3–5 days. These colonies were isolated and purified by restreaming on the same medium (Pérez-Través et al., 2012). The hybrid nature of the colonies selected in MM was confirmed by PCR-RFLP of the genes *UGA3* and *GSY1* to confirm that they showed hybrid profiles (Pérez-Través et al., 2014b).

### Test of Stability

Two strategies were carried out to determine the stability of hybrids: adaptive stabilization by vegetative growth without sporulation and adaptive stabilization by vegetative growth after sporulation. The stability test by vegetative growth was done as described elsewhere (Pérez-Través et al., 2012), with some modifications. The media was a synthetic must with 40 g L^–1^ of glucose, 45 g L^–1^ of fructose, 2.5% of EtOH and 60 mg L^–1^ of potassium metabisulfite, and the experiment was incubated at 28°C. A single colony of each hybrid strain was individually inoculated into 20 mL of this must and they grew in those conditions for 10 days. At that moment, 200 μL of each fermentation, were inoculated in a new fresh media at the same conditions. The process was repeated 5 times. Once the fifth fermentation ended, for each one of the hybrids 10 colonies were tested for their molecular characterization by mtDNA-RFLP and delta elements analysis to be compared with the original hybrid and among them. We considered a genetically stable hybrid when all colonies recovered after individual growths maintained the same molecular pattern than the original culture. Only hybrids that maintained the same molecular pattern in its 10 colonies at the end of the process were considered for the artificial hybrid selection (next section). Only one of the ten colonies of each stable hybrid was randomly selected as a representative for subsequent artificial hybrid selection.

The test of adaptive stability by vegetative growth after sporulation was performed by incubating the hybrids in acetate-agar plates as described in the ‘sporulation assays’ section. For each hybrid, 10 spores were selected and characterized by PCR-RFLP analysis of 4 nuclear genes (*APM3*, *UGA3*, *GSY1*, and *BRE5*) and the internal transcribed spacer (ITS) region to confirm that they still showed a hybrid profile in at least one gene region. These genome regions were tested pairwise and when a hybrid pattern was obtained with the first pair, the next ones were not analyzed. The colonies showing a hybrid profile in at least one region (out of 5) were used for the same adaptive stability test described above for the adaptive stability without sporulation. At the end of the last fermentation, 10 colonies were isolated and they were also tested by mtDNA-RFLP (Querol et al., 1992) and delta elements analysis (Legras and Karst, 2003). Again, only hybrids that showed identical molecular patterns in the 10 derivative colonies at the end of the process were considered for the artificial hybrid selection.

### Artificial Hybrid Selection

Those strains exhibiting a hybrid pattern, according to the different molecular markers used, and were stable during vegetative growth in fermentation without or after sporulation were considered to screen its phenotype for selection. Their growth in the presence of ethanol was monitored by measuring absorbance at 600 nm in a SPECTROstar Omega in SM with 10 g L^–1^ glucose, 20 g L^–1^ fructose, 60 mg L^–1^ potassium metabisulfite and 6.5% of ethanol. Growth conditions and the statistical analysis were performed as described above.

The same ethanol tolerance assay, described above, was performed using these selected hybrid strains, as well as the two parental strains, both at 15 and 25°C. For the enological characterization of the selected artificial hybrids, triplicate fermentations were conducted in 250 mL bottles, closed with Müller valves, containing 200 mL of Verdejo natural must, supplemented with 0.3 g L^–1^ of nutrients, and incubated with shaking (100 rpm) at two different temperatures, 15 and 25°C. The parental strains AJ4 and BMV58 were also included for comparative purposes. Fermentations were followed by weight loss as in Pérez-Través et al. (2014a). At the end of fermentation, supernatant samples were analyzed by HPLC to determine the amount of residual sugar (glucose and fructose), glycerol, ethanol, and organic acids. For this purpose, a Thermo chromatograph (Thermo Fisher Scientific, Waltham, MA), equipped with a refraction index detector, was used. The column employed was a HyperREZTM XP Carbohydrate H + 8μm (Thermo Fisher Scientific) and it was protected by a HyperREZTM XP Carbohydrate Guard (Thermo Fisher Scientific). The conditions used in the analysis were as follows: eluent, 1.5 mM sulfuric acid; flux, 0.6 mL min^–1^; and oven temperature, 50°C. Samples were 5-fold diluted, filtered through a 0.22-μm nylon filter (Symta, Madrid, Spain) and injected in duplicate.

Weight loss data was corrected with respect to the percentage of consumed sugars (Pérez-Través et al., 2014a). Percentages of consumed sugars over time were fitted to a Gompertz equation (Zwietering et al., 1990), and the following kinetic parameters were calculated from the adjusted curves: *m*, maximum sugar consumption rate (g L^–1^ h^–1^); *l*, latency or lag phase period (h); and t50 and t90, time to consume 50% and 90% of sugars (h), respectively. All the data were tested to find significant differences among them by using the one-way ANOVA module of the Statistica 7.0 software (StatSoft, Tulsa, OK, United States). Means were grouped using the Tukey HSD test (α = 0.05).

### Genome Sequencing, Assemblage, and Annotation

Total DNA from the artificial hybrid strain and from the S. *cerevisiae* parental strain AJ4 were extracted according to Querol et al. (1992) and sequenced using the Illumina Miseq system, with paired-end reads of 250 bp (NCBI accession number SRP148850). The genome of Velluto BMV58^TM^, the other parental strain, was already sequenced, assembled, and annotated in a previous study from our lab (Macías et al., in preparation).

Sequencing reads were trimmed and quality filtered using Sickle (Joshi and Fass, 2011), and then assembled following a semiautomatic pipeline (Macías et al., 2019; Morard et al., 2019) that uses programs Velvet (Zerbino and Birney, 2008), Sopra (Dayarian et al., 2010), SSPACE (Boetzer et al., 2011), Gapfiller (Boetzer et al., 2012) and MUMMER (Kurtz et al., 2004). The assembly was confirmed by comparison with that of the reference *S. uvarum* strain CBS 7001 (Scannell et al., 2011).

Genes were annotated combining the *ab initio* method with Augustus (Stanke and Morgenstern, 2005) and annotation transfer method with RATT (Otto et al., 2011). Genes were manually curated using Artemis (Rutherford et al., 2000), NCBI BLAST web interphase (Johnson et al., 2008) and the SGD Database^1^ (Macías et al., 2019; Morard et al., 2019).

### Flow Cytometry Analysis

The DNA contents of the selected hybrid and the parental strains were assessed by flow cytometry using a FACSVerse^TM^ flow cytometer (BD Biosciences). Cells were grown overnight in GPY and 1 OD_600_ of each culture was harvested by centrifugation. DNA staining was performed using dye SYTOX Green (Haase and Reed, 2002). Fluorescence intensity was compared with a haploid (S288c) and diploid (FY1679) reference *S. cerevisiae* strains.

### Copy Number Variation Analysis

The *S. cerevisiae* reads were mapped against the reference genomes of S288c using Bowtie2 version 2.3.2 (Langmead and Salzberg, 2012). Genome annotations were visualized using Artemis (Rutherford et al., 2000) with the mapped reads to predict deletions and duplications present in the *S. cerevisiae* parental. Artificial hybrid reads were mapped to a combination of the *S. cerevisiae and S. uvarum* parental consensus sequences, including mitochondrial genomes, by using bowtie2 version 2.3.2 (Langmead and Salzberg, 2012), with the default settings.

SppIDer (Langdon et al., 2018)-was used to visualize the genome composition of the selected hybrid. By using this tool, the reads of the hybrid genome were mapped to the reference genome of its parental *S. cerevisiae* and *S. uvarum* strains.

Bedtools (Quinlan and Hall, 2010) was used to obtain the coverage “per base”. These coverage files were processed to reduce the noise using a sliding windows method with a window size of 1000 positions. As a complementary approach, CNVnator was used for copy number variation discovery (Abyzov et al., 2011).

### Variant Calling Analysis

The gdtools command installed as part of breseq (Barrick et al., 2014; Bernstein and Carlson, 2014) was used to identify single nucleotide polymorphisms (SNPs) on Genome Diff files. The minimum polymorphism frequency to call an SNP using breseq was set to 0.20. To calculate heterozygosity levels, the SNP number was divided by the genome size of each strain. We subtracted and annotated SNP regions that were not present in the parental genomes but present in the hybrid genome, with an in-house python script.

### RNA-Seq Analysis

The RNA-seq analysis was performed using the cells collected from the Verdejo must micro vinifications, described above. We used white natural must to avoid RNA degradation due to the oxidation of polyphenols present in red musts. Fermentations were followed by weight loss; kinetic parameters were analyzed as explained above.

Cell samples were collected at two different fermentation time points: at the lag phase (4 h) and at the mid-exponential growth phase (24 h at 25°C and 48 h at 15°C respectively). Cells were harvested by centrifugation and then stored at −80°C. Total RNA was extracted following a protocol based on an initial step of washing with DEPC-treated water and subsequent treatments with phenol-Tris, phenol-chloroform (5:1) and chloroform-isoamyl alcohol (24:1), and finally, a first precipitation with LiCl, and a second with sodium acetate and ethanol. After RNA extraction, we combined equal proportions of RNAs from the two parental strains in the same sample to reduce the number of libraries to sequence. Instead of 36 original RNA extracted samples (3 strains × 2 temperatures × 2-time points × triplicate), we had 24 samples to sequence. These samples were sequenced using the Illumina Hiseq 2000, paired-end reads 75 bases long (NCBI accession number PRJNA473074). Sequence reads were trimmed and quality filtered using Sickle (Joshi and Fass, 2011) (length 50, quality 23) and aligned to a combined concatenated reference of both genomes (AJ4 and BMV58) using bowtie2 version 2.3.2 (Langmead and Salzberg, 2012). Read counts for each gene were obtained using HTSeq-count version 0.9.0 (Anders et al., 2015), with a combination of BMV58 and AJ4 annotations and the mapping files ordered by names. The mapping reads with a quality score lower than 2 or those that aligned in more than one genome position were discarded.

All the samples were split into two files: One containing the *S. cerevisiae* gene counts and the other with the *S. uvarum* gene counts, as we had half of the sample containing hybrid reads and the other half with the merged sequences, which corresponded to the *S. cerevisiae* and to the *S. uvarum* parental strains during fermentation. The data was analyzed by a principal component analysis (PCA) among samples using the DESeq2 package (Anders and Huber, 2010). Read counts for each one of the 48 files were extracted and used for differential expression analyses with the EdgeR package (Robinson et al., 2009). Normalization factors were calculated among reads to scale the raw library sizes, the negative binomial conditional common likelihoods were maximized to estimate a common dispersion value across all genes, and finally, the tagwise dispersion values were estimated by an empirical Bayes method based on weighted conditional maximum likelihood.

Finally, genewise exact tests were computed for differences in the means between two groups of negative-binomially distributed counts, only retrieving a gene if the number of counts in all samples is > 1. Differential expression levels (relative RNA counts) between the different conditions were considered as significantly different with a false discovery rate (FDR) (Benjamini and Hochberg, 1995) at a threshold of 5%. Venn Diagrams were constructed with the number of differential expressed genes for each assayed condition and Gene Ontology (GO) terms were attributed using SGD. Statistical overrepresentation tests for the differentially expressed genes were also performed using Panther Version 14.1 (released 2019-03-12) with default settings (Mi et al., 2019). We retrieved *p*-values and fold enrichment for each GO term. Fold enrichment indicates if the observed gene number for each category in the list is higher than the expected, based on the number of uploaded genes. If > 1, it indicates that the category is overrepresented in our experiment. The *p*-values indicate the probability that the number of genes observed in this category occurred by chance, as determined by our reference list.

## Results

### *S. cerevisiae* Parental Strain Selection According to Ethanol Tolerance

The main objective of the present work is to improve ethanol tolerance in a wine *S. uvarum* strain, Velluto BMV58^TM^ (Lallemand Inc.), by interspecific hybridization. First, we characterized and selected a *S. cerevisiae* parental strain that can complement BMV58 with its high ethanol tolerance. For this, we analyzed the growth in several ethanol concentrations of three industrial *S. cerevisiae* strains, previously selected by Lallemand for its tolerance to ethanol in industrial processes. Accordingly, we confirm that *S. uvarum* strain BMV58^TM^ is the one with the lower non-inhibitory concentration (NIC) and minimum inhibitory concentration (MIC) values, being unable to grow in concentrations that did not affect the growth of the *S. cerevisiae* strains (Table 1). The *S. cerevisiae* strain AJ4 was selected for hybridization because it exhibits the highest NIC and MIC values (11.6% and 18.6%, respectively). The parental strains AJ4 and BMV58 were assayed for their mating competence, with an analysis of their *MAT* locus (Supplementary Figure S1) and both were heterozygous *MATa*/*MATalpha*. Their sporulation efficiency and spore viability was tested both on acetate plates and in the rich GPY liquid medium used for rare mating. As mentioned, no sign of sporulation for *S. cerevisiae* AJ4 was detected in GPY after more than ten days. However, sporulation efficiency in GPY was very low and difficult to observe for BMV58, but a few asci were present. On acetate plates, both strains sporulated with spore viabilities of 75% for AJ4 and > 95% for BMV58. Several dissected spores were also assayed for the *MAT* locus and were heterozygous *MATa*/*MATalpha*, indicating that both parental strains are homothallic (data not shown).

**TABLE 1:** One-way ANOVA analysis for the NIC and MIC parameters of 4 different *Saccharomyces* strains.

**Strain**	**NIC**	**MIC**
AJ4	11.65 ± 0.32^d^	18.56 ± 1.48^c^
AJB	10.03 ± 0.16^c^	13.76 ± 0.19^b^
AJW	8.63 ± 0.45^b^	14.94 ± 0.49^b^
BMV58	5.69 ± 0.9^a^	10.8 ± 1.19^a^

*NIC and MIC parameters were obtained for the *S. uvarum* BMV58 and *S. cerevisiae* AJ4, AJB, and AJW strains in SM + ethanol media at 28°C. Standard deviations were obtained from triplicate experiments. Values followed by different superscript letters, within the same column, are significantly different according to an ANOVA and Tukey HSD tests, α = 0.05.*

### Hybrid Generation and Characterization

Selection procedures of hybrids based on auxotrophic complementation of parental strains is difficult since industrial strains are prototrophic. For this reason, spontaneous auxotrophic mutants for AJ4 (lys2^–^) and BMV58 (trp1^–^) were selected by growth on α-AA and 5-FAA agar plates, respectively. However, no ura^–^ auxotrophs were isolated on 5-FOA plates (Pérez-Través et al., 2012).

A rare-mating approach was used to obtain putative allotetraploid hybrids with the whole-genome content of both parents (Pérez-Través et al., 2012). After the rare-mating process, 15 prototrophic colonies were recovered in the selection media. Eight of them were confirmed to be hybrids by PCR amplification and restriction analysis of *UGA3* and *GSY1* gene regions (Pérez-Través et al., 2014b). Seven out of eight colonies (H3 to H5, H8, H12, H14, and H15) showed a hybrid profile in both genes (data not shown).

These 7 hybrids were subjected to a test of stability by vegetative growth during fermentation. Each hybrid was inoculated into synthetic must during five passages. Once the fifth fermentation ended, we isolated colonies and 10 of them were randomly selected for each hybrid. These colonies were molecularly characterized by mtDNA-RFLP and delta element analysis. The analysis of the hybrids revealed that only the 10 colonies from hybrid H8 showed different delta profiles. For the subsequent phenotypic characterization, one of these 10 colonies of each hybrid, showing the same molecular patter, was randomly selected for each hybrid. From now on, these vegetative stabilized hybrids will be named H3, H4, H5, H12, H14 and H15.

Three of the original hybrids (H3, H4, and H14) were able to sporulate with a sporulation efficiency > 95%. Therefore, they were sporulated and more than 16 asci were dissected. Hybrid spore viabilities were: 76.7%, 53.6% and 39% for H3, H4, and H14, respectively. However, only 10 viable spores were selected for each hybrid. These monosporic derivatives were named after the original hybrid name (H3, H4, or H14) followed by a letter and a number indicating the dissection plate coordinates.

The hybrid nature of the monosporic-derivative strains was analyzed by PCR amplification and subsequent restriction analysis of six gene regions to determine the presence of at least one hybrid pattern. According to this analysis, only 9 monosporic strains, all of them recovered from hybrid H14, showed an interspecific hybrid pattern for at least one the genes assayed (Supplementary Table S1). These monosporic derivative hybrids were also subjected to a test of stability by performing fermentation in synthetic must at 25°C. In the end, 10 colonies from each fermentation were isolated and the genome stability was confirmed using δ elements and mtDNA-RFLP patterns (Querol et al., 1992; Legras and Karst, 2003). All these hybrid monosporic derivatives were stable in their patterns along the fermentation.

### Phenotypic and Enological Characterization of the Artificial Hybrids for the Selection of the Best Suitable Strain

**FIGURE 1:**
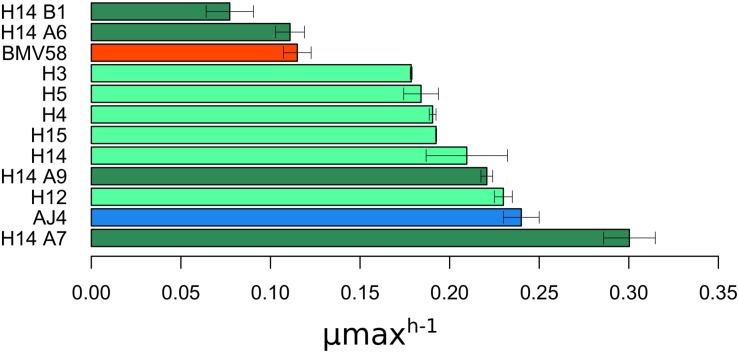
Maximum growth rate (μ max) of the different colonies after stabilization by vegetative growth and sporulation. μ max data is represented as the mean value of three replicates with its standard deviation. The data was retrieved after growing the colonies in SM with 30 g/L of sugars and 6.5%(v/v) of exogenous ethanol. Colonies stabilized by vegetative growth are filled in light green color, and those stabilized by sporulation in dark green; Parental AJ4 is shown in blue, and BMV58 in red.

The strains that showed to be stable during vegetative growth without and after sporulation, along with the two parental strains AJ4 and BMV58, were evaluated for growth in SM (30 g L^–1^ of glucose) supplemented with 6.5% ethanol (Figure 1). We observed that the maximum growth rate varied between the different artificial hybrids and spore derivatives. It is interesting to point out that the monosporic derivative H14A7 showed a higher growth rate, even better than *S. cerevisiae* AJ4. The kinetic parameters for the other strains were intermediate between those of their parents, except H14B1 and H14A6, which show lower maximum growth rates (μ_*max*_). Accordingly, we selected the hybrid spore derivative H14A7 because showed a μ_*max*_ higher than both parents did.

H14A7 was an isolate from a three-spored ascus obtained of H14. Only two of the spores from this ascus were viable (H14A6 and H14A7) (Supplementary Table S1), being one of them, H14A7 the selected strain.

Ethanol tolerance assays of the derivative hybrid were performed at 15 and 25°C using the two parental strains AJ4 and BMV58 as controls. Their NIC and MIC values at both temperatures can be seen in Table 2. H14A7 NIC value at 15°C is the highest, and its MIC values are between both parents at both temperatures.

**TABLE 2:** One-way ANOVA analysis for the NIC and MIC parameters of S. *uvarum* BMV58 and H14A7 strains at 15 and 25°C.

**Strain**	**NIC**		**MIC**	
	**15**°**C**	**25**°**C**	**15**°**C**	**25**°**C**
BMV58	8.93 ± 0.67^a,b^	6.05 ± 0.26^a^	11.86 ± 0.86^a,b^	9.52 ± 0.14^a^
AJ4	6.84 ± 1.26^a,b^	7.97 ± 0,62^a,b^	17.45 ± 1.16^c^	16.73 ± 0.18^c^
H14A7	9.19 ± 0.63^b^	7.56 ± 0.49^a,b^	12.16 ± 0.22^b^	11.44 ± 0.30^b^

*NIC and MIC parameters were obtained for the *S. uvarum* BMV58, *S. cerevisiae* AJ4 and H14A7 *S. cerevisiae* × *S. uvarum* strains in SM + ethanol media at 25 and 15°C. Standard deviations were obtained from triplicate experiments. Values followed by different superscript letters, within the same column, are significantly different according to an ANOVA and Tukey HSD tests, α = 0.05.*

**FIGURE 2:**
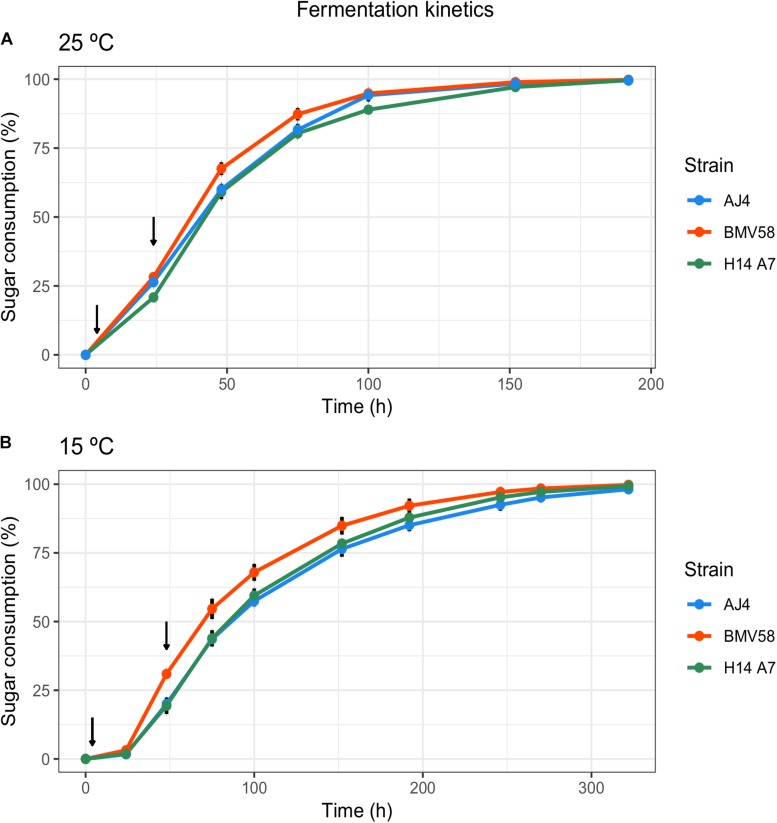
Main analytical and kinetic parameters of the fermentation carried out with both parental strains and the obtained hybrid in Verdejo must at 15 and 25°C. Sugar consumption represents the percentage of sugars consumed at different time points of the fermentation for AJ4 (blue), BMV58(red) and H14A7 (green), at 25°C **(A)** and 15°C **(B)**. Arrows indicate the time points when samples for transcriptomic analysis were taken (*t* = 4 h and *t* = 24 h at 25°C, and *t* = 4 h and *t* = 48 h at 15°C).

Enological properties of the hybrid monosporic derivative H14A7 and the parental strains AJ4 and BMV58 were evaluated by performing fermentations in Verdejo grape musts at 15 and 25°C. Their sugar consumption profiles, kinetic parameters, and metabolite production are shown in Figure 2 and Table 3. Sugars (glucose and fructose) of the Verdejo musts were practically exhausted at the end of all fermentations performed at both temperatures.

**TABLE 3:** Kinetic parameters and chemical composition of fermentation in Verdejo must inoculated with AJ4, BMV58 and H14A7 strains at 15 and 25°C.

	**25**°**C fermentations**			**15**°**C fermentations**		
	**H14A7**	**AJ4**	**BMV58**	**H14A7**	**AJ4**	**BMV58**
		
	**Final must composition**			**Final must composition**		
Glucose (g/L)	0.02 ± 0.02^a^	0.00 ± 0.00^a^	0.03 ± 0.04^a^	0.00 ± 0.00^a^	0.00 ± 0.00^a^	0.00 ± 0.00^a^
Fructose (g/L)	0.77 ± 0.16^a^	1.01 ± 0.44^a^	0.39 ± 0.1^a^	1.41 ± 0.53^a^	2.39 ± 1.01^a^	0.47 ± 0.82^a^
Glycerol (g/L)	11.23 ± 0.13^a^	10.22 ± 0.51^b^	11.66 ± 0.43^a^	8.70 ± 0.09^b^	7.52 ± 0.23^a^	11.07 ± 0.29^c^
Ethanol (%)	12.72 ± 0.36^a^	12.83 ± 0.51^a^	12.38 ± 0.13^a^	12.86 ± 0.12^c^	12.35 ± 0.1^b^	11.69 ± 0.02^a^
Citric acid (g/L)	0.39 ± 0.01^b^	0.27 ± 0.02^a^	0.23 ± 0.02^a^	0.28 ± 0.05^a^	0.29 ± 0,02^a^	0.3 ± 0.01^a^
Tartaric acid (g/L)	2.4 ± 0.12^a^	2.22 ± 0.09^a^	2.19 ± 0.12^a^	1.92 ± 0.09^a^	2.23 ± 0,23^a^	1.88 ± 0.12^a^
Malic acid (g/L)	1.96 ± 0,14^b^	2.68 ± 0.26^a^	1.94 ± 0.22^b^	1.79 ± 0.07^a^	2.66 ± 0,78^a^	1.92 ± 0.11^a^
L- Lactic acid (g/L)	1.02 ± 0.14^b^	1.95 ± 0.31^a^	0.73 ± 0.03^b^	0.38 ± 0.03^a^	0.32 ± 0.02^a^	0.26 ± 0.06^a^
		
	**Kinetic parameters**			**Kinetic parameters**		
		
m (g L^–1^h^–1^)	1.761 ± 0.0985^b^	1.485 ± 0.0706^a^	1.513 ± 0.114^a^	0.78 ± 0.0265^ab^	0.75 ± 0.05^a^	0.924 ± 0.089^b^
l (h)	9.84 ± 0.80194^b^	6.96 ± 0.551^a^	8.081 ± 0.54^a^	23.95 ± 2.17^b^	22.071 ± 1.89^ab^	18.37 ± 0.97^a^
t50 (h)	43.20 ± 1.61^b^	40.88 ± 1.80^b^	36.72 ± 1.37^a^	87.91 ± 4.13^b^	89.89 ± 4.43^b^	73.21 ± 4.13^a^
t90 (h)	93.62 ± 4.44^b^	88.84 ± 4.47^a,b^	77.91 ± 4.29^a^	186.96 ± 7.61^a,b^	207.206 ± 3.22^b^	157.26 ± 3.96^a^
Glycerol/sugar yield (g/g)	0.056 ± 0.0005^a^	0.0512 ± 0.0004^b^	0.058 ± 0.0005^a^	0.044 ± 0.0002^b^	0.038 ± 0.0004^a^	0.055 ± 0.0004^bc^

*m is the maximum sugar consumption rate, l is the fermentation lag phase duration, and t50, t90 are the times employed to consume 50% and 90% of the sugars present in the must. These values were obtained after adjustment to Gompertz equation of three biological replicates. Values are a mean of the three biological replicates followed by their standard deviation. Different superindexes in the same row and belonging to the same temperature are significantly different according to the Tukey HSD test (α = 0.05).*

Glycerol production was higher at 25°C in the H14A7 strain and the *S. uvarum* parental, whereas at 15°C the hybrid derivative showed an intermediate profile of glycerol production, higher than AJ4 but lower than BMV58. The analysis of the production of organic acids showed that parental AJ4 and the hybrid monosporic derivative produce higher amounts of lactic acid compared to BMV58. It is worth to note that H14A7 presented a longer latency phase at both temperatures compared to its parents but, during the exponential phase, exhibited the maximum sugar consumption rate and fermentation speed at 25°C, and an intermediate sugar consumption rate between those of AJ4 and BMV58 at 15°C. Therefore, we can conclude that the hybrid derivative strain inherited the good fermentation performance and the higher production of organic acids from the *S. cerevisiae* AJ4 parent, and the higher synthesis of glycerol from BMV58 (Table 3).

### Comparative Genome Analysis Between the Best Artificial Hybrid and Its Parents

To determine the genome constitution of the artificial hybrid and those changes that occurred during the process of rare-mating hybridization and the subsequent sporulation, a comparative genome analysis between the artificial hybrid derivative and its parents was performed. For this purpose, we sequenced, assembled and annotated the whole genome of monosporic derivative H14A7 and the *S. cerevisiae* AJ4 parental strain. The BMV58 genome sequence and annotation were already available in our laboratory (Macías et al. unpublished data).

A total of 6182 genes of AJ4 were annotated and manually revised. The retrieved BMV58 annotation consists of a set of 5710 manually revised genes. A total of 5393 gene sequences were well annotated and shared between AJ4 and BMV58.

**FIGURE 3:**
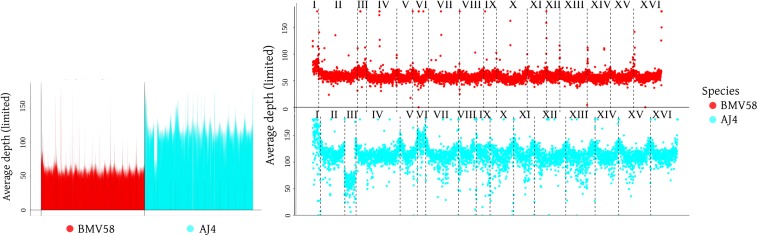
Graphic representation of the hybrid H14A7 genome composition, obtained with sppIDer (https://github.com/GLBRC/sppIDer), a pipeline that uses bwa –mem to map the reads of the hybrid genome to the reference genome of its parental strains, BMV58 and AJ4.

The H14A7 sequence reads were mapped against the genomes of AJ4 and BMV58 strains to unveil its genome constitution by means of an analysis of copy number variations (CNV) in its chromosomes. It is interesting to note that the artificial hybrid H14 and its spore derivative H14A7 inherited the *S. cerevisiae* mitochondrial genome. This genome constitution analysis was complemented with an analysis of ploidy by flow cytometry. Strikingly, although both parents are diploid strains (AJ4, 2.28 ± 0.01; and BMV58, 2.28 ± 0.01), H14A7 is allotriploid (2.98 ± 0.02), and not allodiploid as expected after sporulation of a putative allotetraploid. The analysis of genome sequences confirmed these results and provided more information on the H14A7 genome composition. Average read depths across the *S. cerevisiae* subgenome were twice of the *S. uvarum* subgenome (Figure 3). Together with the flow cytometry results, this suggests that the monosporic derivative H14A7 is allotriploid with a diploid *S. cerevisiae* subgenome and a haploid *S. uvarum* subgenome. An exception was observed for chromosome III, which in both subgenomes appeared in only one copy. These observations were also confirmed by the CNVnator analysis. When we searched for deletions and duplications of small regions with CNVNator, no significant changes were detected.

The *MAT* locus was also tested for strain H14A7, containing a *MATa* allele in the monosomic *S. cerevisiae* chromosome III and a *MATalpha* allele in the haploid *S. uvarum* subgenome (Supplementary Figure S1).

To better understand how the selected spore-derivative hybrid could be originated, we compared single nucleotide polymorphisms (SNPs) in H14A7, AJ4, and BMV58 (Supplementary Table S2). The heterozygosity levels are higher in the *S. cerevisiae* parental strain (0.067% SNPs in the genome) than in the *S. uvarum* one (0.022% SNPs). The hybrid *S. cerevisiae* subgenome maintains the same levels of heterozygosity than AJ4 for each chromosome pair, except for the homozygous chromosome III, due to the single copy maintained in the hybrid. Apart from the SNPs located in chromosome III, H14A7 only showed the fixation of three non-synonymous homozygous SNPs, present in its parental *S. cerevisiae* strain in the form of heterozygous sites, likely by a loss of heterozygosity mechanism. These three homozygous SNPs occurred in gene *TRK2* (YKR050W), located on chromosome XI, which is part of the Trk1p-Trk2p potassium transport system.

### Comparative Expression Analysis During Wine Fermentation

**FIGURE 4:**
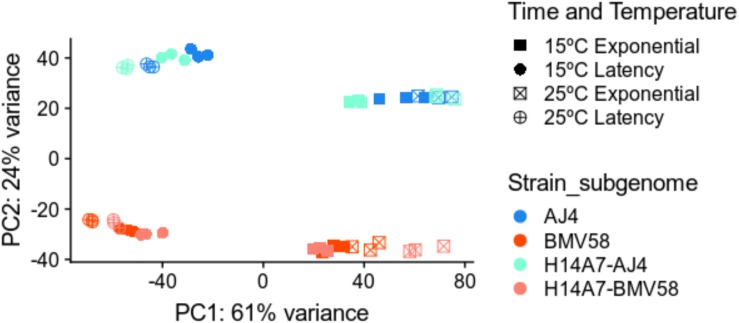
Principal component analysis of the transcriptome variation in *S. uvarum* BMV58, *S. cerevisiae* AJ4, and the *S. uvarum* and *S. cerevisiae* subgenomes of the artificial hybrid under two different temperatures and fermentation phases. All sequenced transcriptomic samples were plotted in this PCA. The PCA plot shows the greater variation in the fermentation phase and in the species gene expression. Triplicates are shown in the same color and shape, as follows: blue, AJ4; red, BMV58; orange, H14A7-*uvarum*; turquoise, H14A7-*cerevisiae*; squares, exponential phase; circles, latency phase; filled, 15°C; a cross, 25°C.

To better understand the properties acquired by the hybrid respect to both parents, we performed a comparative study of gene expression during Verdejo must fermentations. A total number of 36 samples (3 strains × 2 times × 2 temperatures × triplicates) of RNA were retrieved during the fermentations and sequenced. In the case of the artificial monosporic derivative H14A7 samples, transcriptomic data of the *S. uvarum* and *S. cerevisiae* genes were treated separately. A principal component analysis (PCA) with the DESeq2 package was performed. Figure 4 showed that triplicates group together and that the greater variance (61%) in the samples correspond to the fermentation phase variable. The first principal component (PC1) separated samples from latency and exponential growth phases. The second component (PC2), which explains 24% of the variance, is the subgenome (*S. cerevisiae* or *S. uvarum).* The PCA also showed clustering of samples into 4 separate groups: samples belonging to *S. cerevisiae gene* expression in the exponential phase; *S. cerevisiae* gene expression in the latency phase; S. *uvarum* gene expression in exponential phase; and *S. uvarum* gene expression in latency phase.

**FIGURE 5:**
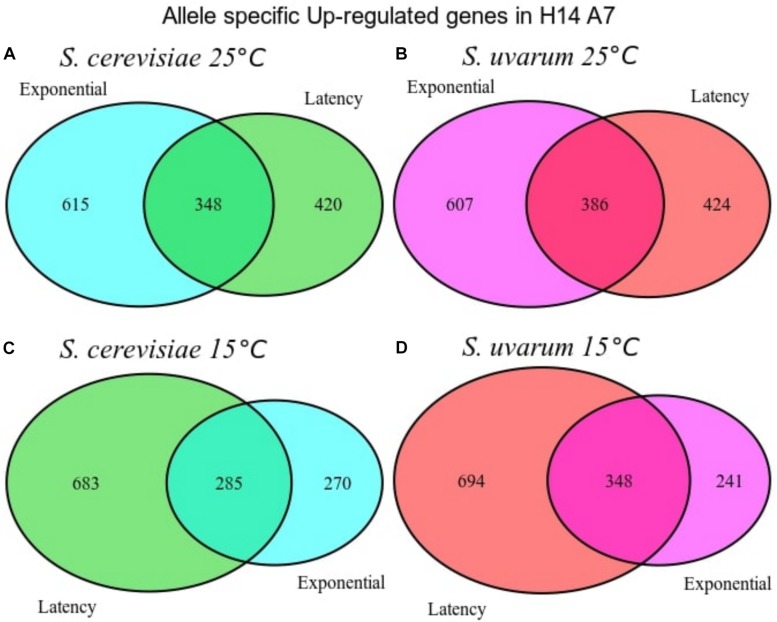
Venn diagrams with the number of differentially expressed genes when performing differential expression analysis between the *S. cerevisiae* and *S. uvarum* subgenomes of the H14A7 monosporic derivative. Up-regulated genes in *S. cerevisiae* genome *against S. uvarum* subgenome at 25°C at two phases **(A)**, Up-regulated genes in *S. uvarum* genome against *S. cerevisiae* subgenome at 25°C at two phases **(B)**, up-regulated genes in *S. cerevisiae* genome against *S. uvarum* subgenome at 15°C at two phases **(C)**, up-regulated genes in *S. uvarum* genome against *S. cerevisiae* subgenome at 15°C at two phases **(D)**.

**FIGURE 6:**
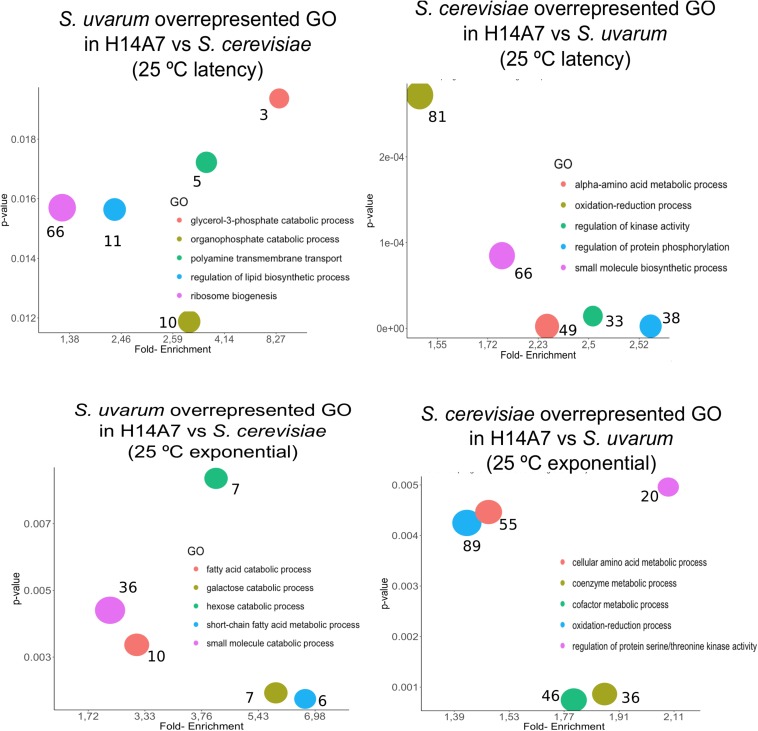
Top 5 significant GO terms retrieved from the differentially expressed genes between the *S. cerevisiae* and *S. uvarum* subgenomes in the H14A7 monosporic derivative at 25°C. For each of the 4 graphs (*S. uvarum* latency overrepresented, *S. cerevisiae* latency overrepresented, *S. uvarum* exponential overrepresented and *S. cerevisiae* exponential overrepresented) the *x*-axis represents de fold-enrichment and the *y*-axis the *p*-value, retrieved from Panther Gene List Analysis. The sizes of the circles represent the number of terms that are included in each GO.

**FIGURE 7:**
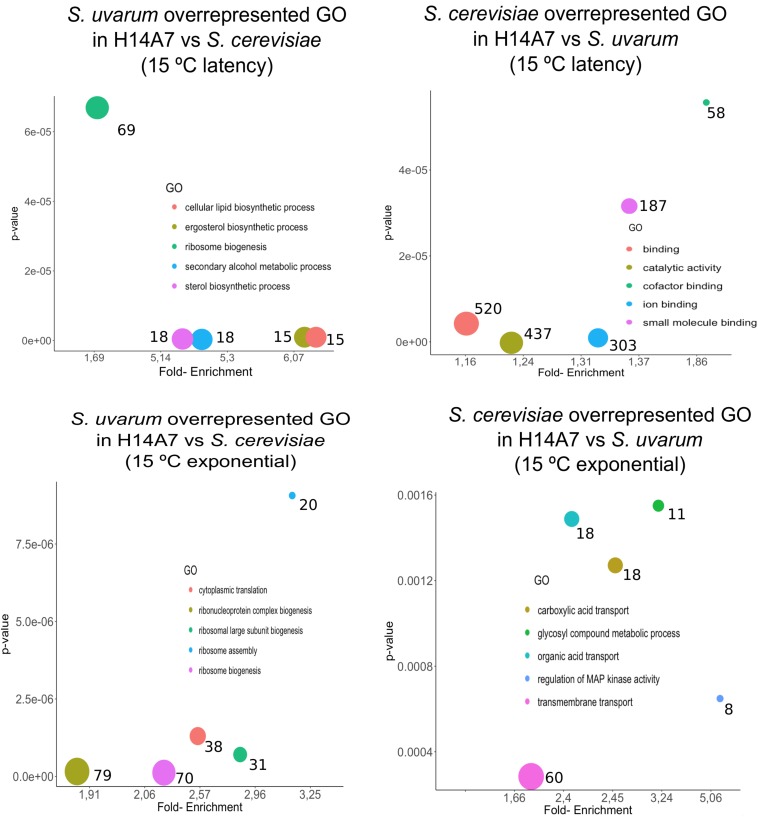
Top 5 significant GO terms retrieved from the differentially expressed genes amongst *S. cerevisiae* and *S. uvarum* subgenomes in the H14A7 monosporic derivative at 15°C. For each one of the 4 graphs (*S. uvarum* latency overrepresented, *S. cerevisiae* latency overrepresented, *S. uvarum* exponential overrepresented and *S. cerevisiae* exponential overrepresented) the *x*-axis represents de fold-enrichment and the *y*-axis the *p*-value, retrieved from Panther Gene List Analysis. The sizes of the circles represent the number of terms that are included in each GO.

We conducted a first differential expression analysis using only the samples corresponding to H14A7 fermentations to compare *S. cerevisiae* and *S. uvarum* gene-specific expressions in this hybrid. We performed simple assays comparing gene expression between the hybrid subgenome genes in 4 conditions (the latency phase at 15 and at 25°C, and the exponential phase at 15 and at 25°C), with adjusted *p*-values < 0.01 (FDR). We normalized *S. cerevisiae* and *S. uvarum* subgenome expressions according to the number of copies of each gene present in the hybrid. Gene-specific overexpression differences can be seen in Figure 5 and the lists of overexpressed genes (with the log2 and the *p*-values) in Supplementary Table S3. At 15°C, the number of differentially expressed genes was higher in the latency phase than in the exponential stage, whereas at 25°C both phases showed a similar number of differentially expressed genes in the hybrid. A GO term enrichment analysis was performed, and the 5 GO terms with a lower *p*-value for each condition are represented against their fold-enrichment in Figures 6, 7.

In the hybrid derivative strain, 348 and 386 *S. uvarum* specific genes were up-regulated at 15 and 25°C, respectively, in both fermentation phases, 217 of them were in common for both temperatures at both phases in comparison with the *S. cerevisiae* gene. Two genes are remarkable from an enological point of view: *ATF2* (YGR177C), encoding an acetyl-transferase, which forms volatile esters during fermentation, and *RSB1* (YOR049C), coding for a putative sphingoid long-chain base (LCB) efflux transporter; which has a role in glycerophospholipid translocation related with membrane composition.

At the latency phase of the fermentation at 15°C, 694 genes were up-regulated in the hybrid *S. uvarum* subgenome in comparison to the *S. cerevisiae* allele. In the GO-term enrichment analysis with a BH correction, one pathway was statistically overrepresented because it appeared with a max *p*-value of 0.05: the ergosterol biosynthesis process [GO:0006696], including genes *ERG1*, *ERG3*, *ERG5*, *ERG6*, and *ERG27* (Figure 7). Terms related to secondary alcohol metabolic process [GO:1902652], steroid metabolic process [GO:0008202], cellular lipid biosynthetic process [GO:0097384], and ribosomal large subunit biogenesis [GO:0042273] were also overrepresented.

We also compared gene-specific up-regulation in the *S. cerevisiae* part of the hybrid (Figures 5A,C). The most significant GO terms enrichments, with a maximum *p*-value of 0.05, were obtained for the latency phase at 15°C, in which 683 genes were up-regulated. These 4 enriched molecular functions were cofactor binding, ion binding, catalytic activity and vitamin binding (Figure 7).

It is remarkable that the fatty acid catabolic process and short-chain fatty acid metabolic process are overrepresented terms in the *S. uvarum* subgenome when compared with the *S. cerevisiae* subgenome during the exponential phase at 25°C. These two GO terms could be related to the H14A7 behavior during alcoholic fermentation, as they are related to membrane composition of yeast cells and, thus, to ethanol tolerance.

In the subsequent differential expression analyses, we compared gene expression during fermentation of the *S. cerevisiae* genes of H14A7 monosporic derivative and those from the parental AJ4, and of the *S. uvarum* genes of H14A7 and those from the parental BMV58. We analyzed H14A7 differentially expressed genes against AJ4 (adjusted *p*-values of 0.05) and only found 5 up-regulated genes, including *FSH1*, encoding a serine hydrolase, and *ARG1*, involved in the arginine biosynthesis pathway. Of the 66 down-regulated genes, 36 of them are located on chromosome III, present as a single copy in the hybrid. It is important to remark this under-expression is significant considering that expression levels were corrected according to the number of copies of the genes. Other underexpressed genes in the hybrid are *GPX2*, encoding a glutathione peroxidase; *ARE1*, an acyl-coenzyme A; *NDE2*, a NADH dehydrogenase; and *ADH2*, alcohol dehydrogenase II, which catalyzes the conversion of ethanol to acetaldehyde (Supplementary Table S4).

RNA seq analysis of *S. uvarum* allele expression between H14A7 and BMV58 showed that there were 33 differentially expressed genes (adjusted *p*-values of 0.05): 17 of them are up-regulated in H14A7 and 16 up-regulated in BMV58 (Supplementary Table S4). It is worth noticing that the gene *ADH5*, encoding an alcohol dehydrogenase involved in ethanol synthesis, is overexpressed in the hybrid derivative H14A7. The function of *ADH5* is uncharacterized, though it has been proposed to share a common ancestor with ADH1/ADH2, from which it appeared to have diverged as part of a whole-genome duplication occurred in the ancestor of the *Saccharomyces* lineage (Wolfe and Shields, 1997).

**FIGURE 8:**
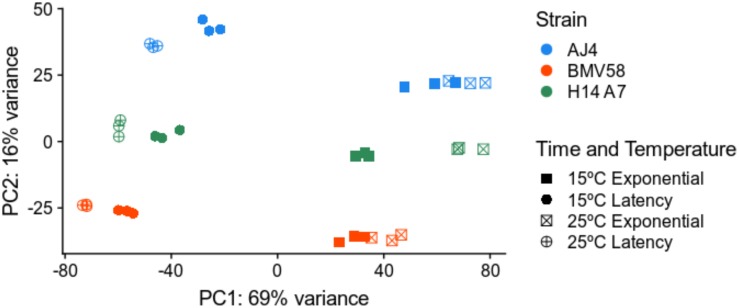
Principal Component Analysis of the transcriptome variation in *S. uvarum* BMV58, *S. cerevisiae* AJ4, and the monosporic derivative H14A7 genome (*S. uvarum* + *S. cerevisiae* subgenomes) under two different temperatures and fermentation phases. All sequenced transcriptomic samples were plotted in this PCA (3 strains × 2 phases × 2 temperatures × triplicates). The PCA plot shows the greater variation in expression levels in the fermentation phase and in the species-specific gene expression. Triplicates are shown in the same color and shape, as follows: blue, AJ4; red, BMV58; green, H14A7; squares, exponential phase; circles, latency phase; filled, 15°C; a cross, 25°C.

As a complementary approach to compare AJ4 and BMV58 parental strains with H14A7 gene expression, we also compared each gene expression of the parental (AJ4 and BMV58, respectively) with the total addition of the *S. cerevisiae* and *S. uvarum* alleles expression of the hybrid. With this approach, we could compare the whole genome expression of the hybrid with the expressions of each parent. We found more significant differentially expressed genes than in the subgenome comparisons. A PCA analysis that groups samples according to their gene expressions can be seen in Figure 8. Differentially expressed gene lists and the complete GO and pathway enrichment terms are available in Supplementary Table S5. Supplementary Figures S2–S5 also depict the number of genes belonging to each GO term.

At the latency phase of fermentations at 25°C, the hybrid showed up-regulation in amino acid biosynthesis when compared with both AJ4 and BMV58 strains, in 46 and 43 genes, respectively (Supplementary Figures S2, S4). Genes *ARO1*, *ARO80*, and *HIS2* are more expressed in H14A7 than in BMV58, *CYS3, MET8*, and *TRP2* are more expressed in H14A7 with respect to AJ4, and *HIS1*, *MET6*, and *ARO8* are up-regulated in comparison to both parents.

At the exponential phase during fermentations at 25°C, H14A7 showed an up-regulation in oxidative-reduction processes with respect to BMV58, and in glycogen biosynthesis, galactose degradation and hexose catabolism in comparison with AJ4. At the latency phase during fermentations at 15°C, the hybrid derivative overexpressed the ribosome biosynthesis genes in comparison with AJ4, and transmembrane transport genes and genes that respond to oxidative stress with respect to BMV58. Finally, at the exponential phase at 15°C, the hybrid overexpressed alpha-amino acid metabolism genes in comparison to BMV58 and ergosterol and sterol biosynthesis genes in comparison to AJ4.

It has to be mentioned that, during the exponential phase, the GO terms: positive regulation of ergosterol biosynthetic process, positive regulation of steroid biosynthetic process, positive regulation of steroid metabolic process, and positive regulation of sterol biosynthetic process, are over-represented in the genome expression of H14A7 against AJ4 at 15°C, and the GO term: positive regulation of alcohol biosynthetic process, at 25°C. At 15°C during the exponential phase, the GO terms: alpha-aminoacid metabolic process and cellular amino acid metabolic process are among the overrepresented GO terms from the differentially expressed genes between H14A7 and BMV58.

As a short summary, *S. cerevisiae* and *S. uvarum* alleles are differentially expressed in H14A7. This differential expression among alleles is very evident in the latency stage at 15°C, genes involved in the ergosterol biosynthetic process and in cellular lipid biosynthetic process are overexpressed in the *S. uvarum* subgenome, whereas the *S. cerevisiae* subgenome overexpressed genes are involved in catalytic activities, among others. When comparing H14A7 total genes against AJ4 and BMV58 parents, the most interesting result is the differential expression of genes related to amino acid biosynthesis.

## Discussion

In the last decade, a great effort has been devoted to the study of natural *Saccharomyces* hybrids present in industrial fermentations (Kodama et al., 2005; Peris et al., 2018). These *Saccharomyces* hybrids have mainly been isolated from fermentative environments in European regions with Continental and Oceanic climates, and they were originated by spontaneous hybridization between *S. cerevisiae* and a cryophilic species: *S. eubayanus*, *S. kudriavzevii*, or *S. uvarum* (Boynton and Greig, 2014). The best-known example is the lager yeasts *S. pastorianus*, a hybrid between *S. cerevisiae* and *S. eubayanus* (Monerawela and Bond, 2017).

The physiological characterization of natural hybrids demonstrated that they inherited the good fermentation performance and ethanol tolerance of *S. cerevisiae* and the ability to grow at lower temperatures of the *S.* non-*cerevisiae* partner, as well as other properties of enological interest (Pérez-Torrado et al., 2018). These interesting properties contributed by the *Saccharomyces* non-*cerevisiae* species prompted the development of artificial interspecific hybrids for industrial applications. The main purpose was the generation of new hybrids to increase diversity, such as in the case of lager yeasts (Hebly et al., 2015; Mertens et al., 2015), or to improve low-temperature tolerance to wine strains (Kishimoto, 1994; Origone et al., 2018; García-Ríos et al., 2019). However, the main purpose of this study is to obtain an artificial *S. cerevisiae* × *S. uvarum* hybrid conjugating the interesting enological properties of a commercial wine *S. uvarum* strain, and the high ethanol tolerance of a *S. cerevisiae* strain, able to grow at ethanol concentrations in which most of the *Saccharomyces* yeasts are not able to grow (Arroyo-López et al., 2010).

It has been reported that increased genome size on the hybrids can confer adaptive flexibility fermenting under different conditions (Miklos and Sipiczki, 1991; Pfliegler et al., 2014) and in the case of our hybrid derivative strain, that proved to be true.

Ploidy of hybrids influences fermentation performance, a triploid strain, as in our case, is improving fermentation when compared with diploid strains (Krogerus et al., 2016). This effect was more remarkable when fermentation took place at 25°C, in which maximum growth rate of the hybrid was higher than the parental rates, but also at 15°C, in which the hybrid showed an intermediate behavior, as described for other *S. cerevisiae* × *S. uvarum* hybrids (Coloretti et al., 2006).

Artificial hybrids are usually obtained by ‘canonical’ mating between haploid cells/spores of opposite mating types, either by spore-to-spore crosses or by mass mating between haploid spores/cells (Zambonelli et al., 1997; Caridi et al., 2002; Antunovics et al., 2005). However, the genomic characterization of natural *S. cerevisiae* × *S. kudriavzevii* hybrids (Morard et al., 2020) suggests that, in these hybrids, the most probable mechanism of hybridization is ‘rare’ mating, although not the only one. Diploid *Saccharomyces* cells can become mating competent by a mating-type conversion to a homozygous genotype (Gunge and Nakatomi, 1972), being able to cross with another mating-competent haploid or diploid cell. This technique, known as rare mating, is less common because hybridization frequency is lower (‘rare’) than those obtained by spore-to-spore or mass-mating conjugations. However, as hybrid genomic architectures will differ according to the mating involved in the hybridization, rare mating has the advantage of maintaining the heterozygosity levels of the parents in all the initial putative allotetraploid hybrids (Pérez-Través et al., 2012; Bellon et al., 2015). The first step required for rare mating is the selection of natural auxotrophic markers in the strains to hybridize, so the prototrophic recovery technique can be used to select the hybrids.

Theoretically, when diploid strains are used to obtain hybrids, as in the case of our *S. cerevisiae* and *S. uvarum* selected parental strains, allotetraploids with the same putative genomic constitution of the parents are obtained. If a hybrid strain is going to be transferred to the industry, it is necessary to ensure its genomic stability. Then, an adaptive stability test needs to be performed. In our case, it was carried out by vegetative growth in fermentative conditions, mimicking the winemaking process, for hybrids and spore-derivative hybrids. During the mitotic or meiotic divisions, different genomic rearrangements or chromosome segregations can be produced, giving rise to a variety of derived allopolyploids (during vegetative growth) and allodiploids (after sporulation) and even mosaic strains with potential physiological differences of interest. An autotetraploid produces autodiploid spores possessing two complete sets of chromosomes, but malsegregation of the octavalent chromosomes during meiosis usually results in aneuploidies. An allotetraploid also produces diploids but these are not autodiploids but allodiploids due to the phenomenon referred to as autodiploidization of the allotetraploid meiosis (Karanyicz et al., 2017). If we take into account all the obtained hybrids, the different behavior and genome composition can be due to different factors considered above, and on the other hand, during the stabilization process, we did not use a high selective pressure, so chromosome losses and stabilization can occur in different ways by chance.

Artificial interspecific hybrids are often disadvantageous compared with their parental species because of their potential reduced viability (Mercer et al., 2007; Piotrowski et al., 2012). However, one of the hybrid monosporic derivatives, H14A7, showed hybrid vigor (Lippman and Zamir, 2007). Thus, H14A7 performed wine fermentations at 25°C faster than its parents and the other derived hybrid, and at lower temperatures showed a better behavior than the *S. cerevisiae* parental strain.

As a monosporic derivative of a putative allotetraploid hybrid generated by rare mating, strain H14A7 was expected to be an allodiploid hybrid. However, a combination of flow cytometry and genome sequencing data indicated that H14A7 strain is an almost perfect allotriploid, with one copy of the *S. uvarum* genome, and two heterozygous copies of each *S. cerevisiae* chromosome, except chromosome III, which is present in one copy. Moreover, the levels of heterozygosity of the *S. cerevisiae* subgenome of the hybrid, except for the monosomic chromosome III, were identical to those of the parental *S. cerevisiae* genome. This indicates that the hybrid maintains the two homologous copies of the *S. cerevisiae* parental chromosomes, with the exception of chromosome III.

There are two possible explanations for the genome composition of this monosporic-derivative hybrid H14A7. In the first, the original hybrid H14 could be a perfect allotetraploid, and the missegregation of the homologous *S. cerevisiae* chromosomes during the meiotic division I generated the H14A7 allotriploid. The different meiotic behavior of the subgenomes is consistent with the autodiploidization of the allotetraploid meiosis (Sipiczki, 2018). This scenario is supported by previous studies with artificial *S. cerevisiae* × *S. uvarum* hybrids (García-Ríos et al., 2019). Allotetraploids are more prone to malsegregation than the autotetraploids, supposedly due to occasional allosyndetic pairing between homeologous chromosomes of the subgenomes (Sipiczki, 2018). In H14A7, it could be hypothesized that the *S. cerevisiae* subgenome, as a whole, failed to perform normal meiosis I. Another scenario, which could have produced an allotriploid from an allotetraploid, is the loss of the *S. uvarum* part of the hybrid during the course of successive meiotic divisions (Antunovics et al., 2005), a process termed genome autoreduction in meiosis (GARMe) (Sipiczki, 2018). This scenario is less relevant here because it takes place after the breakdown of the sterility barrier and cannot result in a one-step malsegregation of all chromosomes of the *S. uvarum* subgenome.

In the second hypothesis about the origin of the H14A7 monosporic derivative, the original hybrid H14 could be originated by a rare mating event between a mating-competent *S. cerevisiae* diploid cell and a *S. uvarum* haploid cell. The subsequent sporulation of this allotriploid, the two *S. cerevisiae* homologous chromosomes and the *S. uvarum* homeologous one should move together during the wrong meiosis I division. In this case, two spores would be viable and the other two non-viable, which is congruent with the tetrad composition from which the H14A7 spore was dissected. This scenario is supported by a previous study in our laboratory, in which an artificial *S. cerevisiae* × *S. kudriavzevii* hybrid was generated by rare mating (Morard et al., 2020), This *S. cerevisiae* × *S. kudriavzevii* hybrid showed the same genome composition than H14A7, it was an allotriploid with one copy of the non-*cerevisiae* (in this case, *S. kudriavzevii*) genome and two heterozygous copies of each *S. cerevisiae* chromosome (the same than its *S. cerevisiae* parental strain T73), except a monosomic chromosome III.

Both parental *S. kudriavzevii* (CR85) and *S. uvarum* (BMV58) strains were able to sporulate in the rare-mating rich media, although the first much more efficiently than the latter. The most important difference between both studies is the fact that the artificial *S. cerevisiae* × S. *uvarum* hybrid H14 was subjected to sporulation to generate H14A7, but the artificial *S. cerevisiae* × *S. kudriavzevii* hybrid not, but in both cases converged to analogous genome compositions.

Therefore, the genome composition of H14A7 indicates that the original hybrid H14 could be the result of a “rare mating” event involving a mating-competent *S. cerevisiae* AJ4 diploid cell and a *S. uvarum* BMV58 haploid or mating-competent diploid cell with the opposite mating type.

However, our spore-derivative hybrid resulted to be an aneuploid allotriploid with one *S. uvarum* genome copy, and two heterozygous copies of each *S. cerevisiae* chromosome, with the exception of a single copy of chromosome III, which contains the *MAT* locus. This result opens the possibility that the parental diploid *S. cerevisiae* strain acquired mating-competence, not by becoming homozygous for the MAT locus due to gene conversion, but because of the loss of one of the chromosomes III. A mating-competent diploid *S. cerevisiae* cell, monosomic for chromosome III with the *MATa* allele, could conjugate with a *MATalpha* haploid or *MATalpha*/*MATalpha* diploid cell of *S. uvarum* to generate H14. This scenario is supported by the fact that the artificial *S. cerevisiae* × *S. kudriavzevii* hybrid generated by rare mating (Morard et al., 2020), but not subjected to sporulation, also was an allotrianeuploid with one copy of the *S. kudriavzevii* genome and two highly heterozygous copies of each *S. cerevisiae* chromosome, except a monosomic chromosome III. This is also congruent with the fact that the *S. cerevisiae* chromosome III, one of the smallest, shows the highest loss frequency (Kumaran et al., 2013), and the fact that the presence of a single copy of chromosome III in diploid hybrid sub-genomes is common in rare mated hybrids (Krogerus et al., 2016).

However, as the genome sequence of the original hybrid H14 is not available, we cannot completely discard that the rare mating, originating H14, could involve a *S. cerevisiae* euploid cell competent for mating due to gene conversion. In that case, the presence of only one *S. cerevisiae* chromosome III copy in H14A7 could be the result of a chromosome loss during the meiotic division of the H14 hybrid, as chromosome III is one of the least stable chromosomes also in alloploid hybrid genomes (Kumaran et al., 2013). In other words, as the genome composition of H14 is unknown, we cannot determine if the lack of one copy of the *S. cerevisiae* chromosome III in H14A7 is due to a prezygotic (occurring in AJ4, the *S. cerevisiae* parent, before the hybridization) or to a postzygotic (taking place during the meiotic division of the hybrid cell) event.

The availability of artificial hybrids, in addition to their biotechnological interest, offers new challenges to study how two genomes, two transcriptomes, two proteomes, and two metabolomes interact to merge into a single system in the hybrid, and what are the consequences of this fusion to generate functional innovations for the adaptation to wine fermentation environments. In our case, we analyzed transcriptomic data obtained during fermentation at two temperatures, 15°C typical for white and rosé wines, and 25°C for red wines. Multivariate analysis showed that the first two principal components, corresponding to the fermentation phase and species, respectively, described 84% of the variability. This result corroborates that strain behavior depends strongly on the wine fermentation phase (Varela et al., 2005; Zuzuarregui et al., 2006; Marks et al., 2008) and on the properties of each strain (James et al., 2003; Tronchoni et al., 2014, 2017). The third factor that affected gene expression was the temperature, mainly due to cold stress response (Tronchoni et al., 2014, 2017).

In the comparative expression analysis between hybrid subgenomes, previous studies (Duval et al., 2010; Pfliegler et al., 2014) reported that each parental fraction act differentially during fermentation; being the *S. cerevisiae* subgenome more efficient in fermentation performance and the *S. uvarum* in temperature adaptation. In our case, we observed the most significant differences in the fermentation latency phase, when yeasts have to cope with the new stress conditions of the beginning of fermentation, such as high osmolarity due to increased sugar concentrations, high sulfite levels, acid stress, and low temperature, in the case of fermentation at 15°C. At this temperature, whilst the *S. cerevisiae* hybrid subgenome focuses on catalytic activity and nutrient uptake (cofactor, ion, and vitamin binding), congruent with its better nutrient uptake efficiency (Alonso-del-Real et al., 2019), *S. uvarum* fraction of the hybrid shows a higher expression in ribosome biogenesis, involved in the translation machinery necessary for growth and division, as well as in the metabolism of ergosterol, a membrane compound required for membrane protein trafficking at low temperature (Parks et al., 1995; Abe and Minegishi, 2008). An analysis of the differential expression between *S. cerevisiae* and *S. kudriavzevii*, another cryophilic species, during fermentation at low temperature, concluded that *S. kudriavzevii*, under cold stress, enhances translation efficiency by synthesizing ribosomes to overcome the alteration in the stability of functional RNAs (Tronchoni et al., 2014). This response to low temperature was also observed in a transcriptome analysis of natural *S. cerevisiae* × *S. kudriavzevii* hybrids (Tronchoni et al., 2017), in which, as occurs in our *S. cerevisiae* × *S. uvarum* hybrid, the most remarkable group of upregulated genes corresponded to the translation machinery category and membrane composition due to the response of the non-*cerevisiae* subgenome to cope with the cold shock.

In the latency phase of the fermentation at 25°C, the *S. uvarum* subgenome showed two up-regulated genes, *GPD1* and *GPD2*, of great importance because they encode glycerol-3-phosphate dehydrogenases involved in glycerol synthesis. The higher production of glycerol, typical of cryophilic species such as *S. uvarum* and *S. kudriavzevii*, has been proposed as a mechanism to adapt to low-temperatures, high osmolarity, and also to maintain the NAD + /NADH redox balance during fermentation (Oliveira et al., 2014; Pérez-Torrado et al., 2016). According to these results, we can conclude that the interactions between the two subgenomes in the hybrid improve those differential species-specific adaptations to the wine fermentation environments, already present in the parental species.

Regarding the ethanol tolerance of H14A7, which proved to be higher than BMV58 but lower than AJ4 at the tested temperatures, it is difficult to analyze specific gene expression, as yeast answer to ethanol stress is complex and not fully understood yet (Mager and Moradas Ferreira, 1993). However, there are some traits that have been related to ethanol tolerance answer: changes in membrane composition, as unsaturated fatty acid and ergosterol content (Mishra and Prasad, 1989; Vanegas et al., 2012), and different amino acid presence in media (Hirasawa et al., 2007).

When we compared GO term over-representation in *S. uvarum* and *S. cerevisiae* subgenomes of the hybrid that could be related to ethanol tolerance, we focused on transcriptomic data obtained in the exponential phase because, during the latency phase, the amount of ethanol in the media is low. In H14A7, some of the GO terms of genes that are differentially regulated in the species subgenomes of the hybrid, are fatty acid catabolic process and short-chain fatty acid metabolic process (*S. uvarum* vs. *S. cerevisiae* exponential 25°C) as well as cellular amino acid metabolic process (*S. cerevisiae* vs. *S. uvarum* exponential 25°C). The two first processes are related to membrane composition modification as a response to the effect of the ethanol on membrane fluidity (Ma and Liu, 2010). Our results suggest that H14A7 is combining *S. cerevisiae* and *S. uvarum* strategies to respond to ethanol stress.

Nevertheless, this transcriptomic analysis is an attempt to determine the relative contribution of each subgenome in H14A7, but the equilibrium acquired between both subgenomes in the hybrid is the result of complex processes, and some up-regulated genome-specific alleles may be under the control of regulators of the other species (Tronchoni et al., 2017).

## Data Availability Statement

The sequence data files for this study can be found in the NCBI SRA accession numbers SRP148850 and PRJNA473074.

## Author Contributions

ML-P, JG, EB, and AQ conceived and designed the experiments. ML-P, LP-T, and SM-C performed the experiments. JH supported and supervised the industrial application. ML-P, LP-T, SM-C, JG, JH, EB, and AQ analyzed the data and wrote the manuscript. All the authors have seen and approved the present manuscript, they have contributed significantly to different parts of the work.

## Conflict of Interest

The monosporic derivative H14A7 is being commercialized by Lallemand Inc., with the commercial name of Velluto Evolution^TM^. JH is employed by the company Lallemand Bio, S.L. The remaining authors declare that the research was conducted in the absence of any commercial or financial relationships that could be construed as a potential conflict of interest.

## Footnotes

1 http://www.yeastgenome.org/

## Supplementary Material

FIGURE S1 Agarose gel electrophoresis showing the *MAT* locus restriction patterns of the artificial hybrid spore derivative H14A7 and the *S. cerevisiae* AJ4 and *S. uvarum* BMV58 parental strains (indicated in red and blue, respectively). PCR fragments were amplified with *MATa* (amplicon length 544 bp) and *MATalpha* (404 bp) specific primers and digested with endonuclease *Mse*I to differentiate the *MAT* alleles of the parental species. The length of the diagnostic bands, specific of *S. cerevisiae* and *S. uvarum*, are indicated in red and blue, respectively. Restriction fragments were separated on 3% agarose gel in 0.5× TBE buffer and a mixture of 50-bp 100-bp DNA ladder markers was used as size standards (m).

FIGURE S2 Overrepresented GO terms from the differentially expressed genes between H14A7 and AJ4. H14A7 global expression is enriched in these terms compared to AJ4. The number of genes that belong to each GO is represented in the bar in 4 different colors (red for overrepresentation in samples belonging to the exponential phase at 15°C, green for exponential at 25°C, blue for latency at 15°C, and violet for latency at 25°C).

FIGURE S3 Overrepresented GO terms from the differentially expressed genes between AJ4 and H14A7. AJ4 global expression is enriched in these terms compared with H14A7. The number of genes that belong to each GO is represented in the bar in 4 different colors (red for overrepresentation in samples belonging to the exponential phase at 15°C, green for exponential at 25°C, blue for latency at 15°C, and violet for latency at 25°C).

FIGURE S4 Overrepresented GO terms from the differentially expressed genes between H14A7 and BMV58. H14A7 global expression is enriched in these terms compared with BMV58. The number of genes that belong to each GO is represented in the bar in 4 different colors (red for overrepresentation in samples belonging to the exponential phase at 15°C, green for exponential at 25°C, blue for latency at 15°C and violet for latency at 25°C).

FIGURE S5 Overrepresented GO terms from the differentially expressed genes between BMV58 and H14A7. BMV58 global expression is enriched in these terms compared with H14A7. The number of genes that belong to each GO is represented in the bar in 4 different colors (red for overrepresentation in samples belonging to the exponential phase at 15°C, green for exponential at 25°C, blue for latency at 15°C and violet for latency at 25°C).

TABLE S1 PCR-RFLP analysis of 10 colonies isolates from the different hybrids obtained by sporulation.

TABLE S2 Total number of SNPs in AJ4, BMV58 and H14A7 strains.

TABLE S3 Excel spreadsheet containing, in separate sheets, the lists of up-regulated species alleles (*S. cerevisiae* and *S. uvarum* alleles overexpression respectively) in H14A7 transcriptomic samples retrieved under 6 different conditions: Latency phase at 25°C, exponential phase at 25°C, latency phase at 15°C; exponential phase at 15°C; latency phase at both temperatures and exponential phase at both temperatures. These gene lists contain the log2 fold change and the *p*-values of the genes. Gene Ontology terms were retrieved from these genes lists by using SGD and they are also specified in separate sheets.

TABLE S4 Excel spreadsheet containing, in separate sheets, the list of genes that were deferentially expressed among H14A7 *S. uvarum* alleles and BMV58 and H14A7 *S. cerevisiae* alleles and AJ4 during all the fermentation conditions.

TABLE S5 Excel spreadsheet containing, in separate sheets, the lists of up-regulated genes among H14A7 *S. uvarum* + *S. cerevisiae* alleles and BMV58 and H14A7 *S. uvarum* + *S. cerevisiae* alleles and AJ4 strains for 4 different conditions: Latency phase at 25°C, exponential phase at 25°C, latency phase at 15°C and exponential phase at 15°C. Gene Ontology term and pathway enrichment were retrieved from these genes lists by using SGD and they are also specified in separate sheets.
