# Supplementary figures and images for: Differential Contribution of the Parental Genomes to a S. cerevisiae × S. uvarum Hybrid, Inferred by Phenomic, Genomic, and Transcriptomic Analyses, at Different Industrial Stress Conditions
Source: Front Bioeng Biotechnol. 2020 Mar 3;8:129. doi: 10.3389/fbioe.2020.00129 (PMC7062649; doi:10.3389/fbioe.2020.00129)

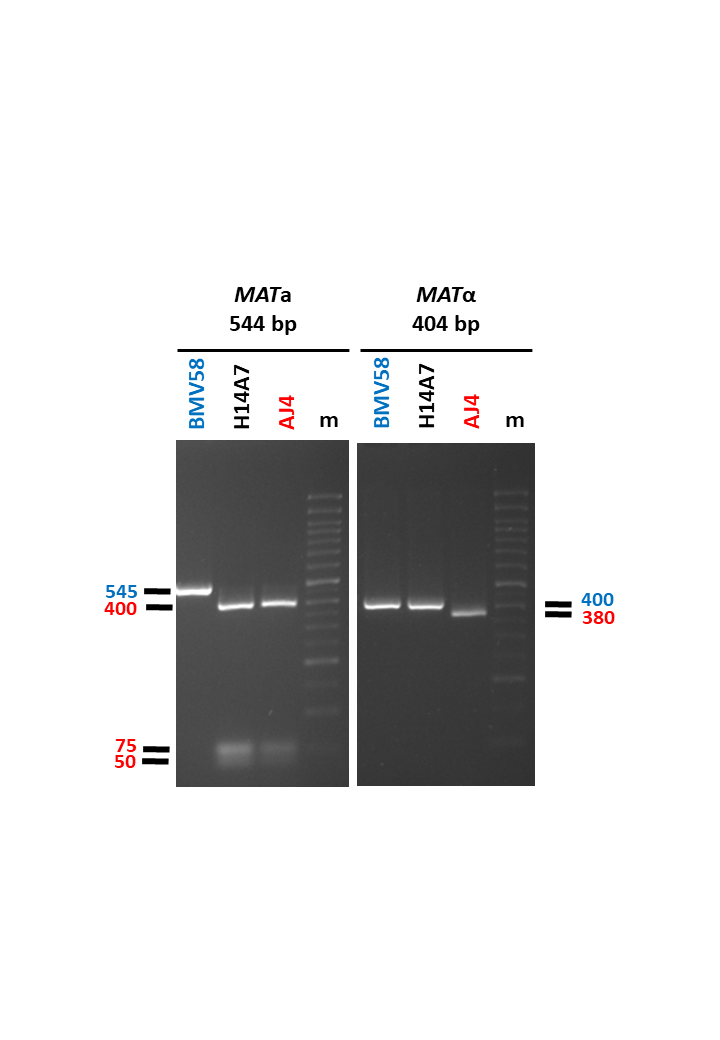

Supplement: FIGURE S1 — Agarose gel electrophoresis showing the MAT locus restriction patterns of the artificial hybrid spore derivative H14A7 and the S. cerevisiae AJ4 and S. uvarum BMV58 parental strains (indicated in red and blue, respectively). PCR fragments were amplified with MATa (amplicon length 544 bp) and MATalpha (404 bp) specific primers and digested with endonuclease MseI to differentiate the MAT alleles of the parental species. The length of the diagnostic bands, specific of S. cerevisiae and S. uvarum, are indicated in red and blue, respectively. Restriction fragments were separated on 3% agarose gel in 0.5× TBE buffer and a mixture of 50-bp 100-bp DNA ladder markers was used as size standards (m). [file Image_1.TIF]

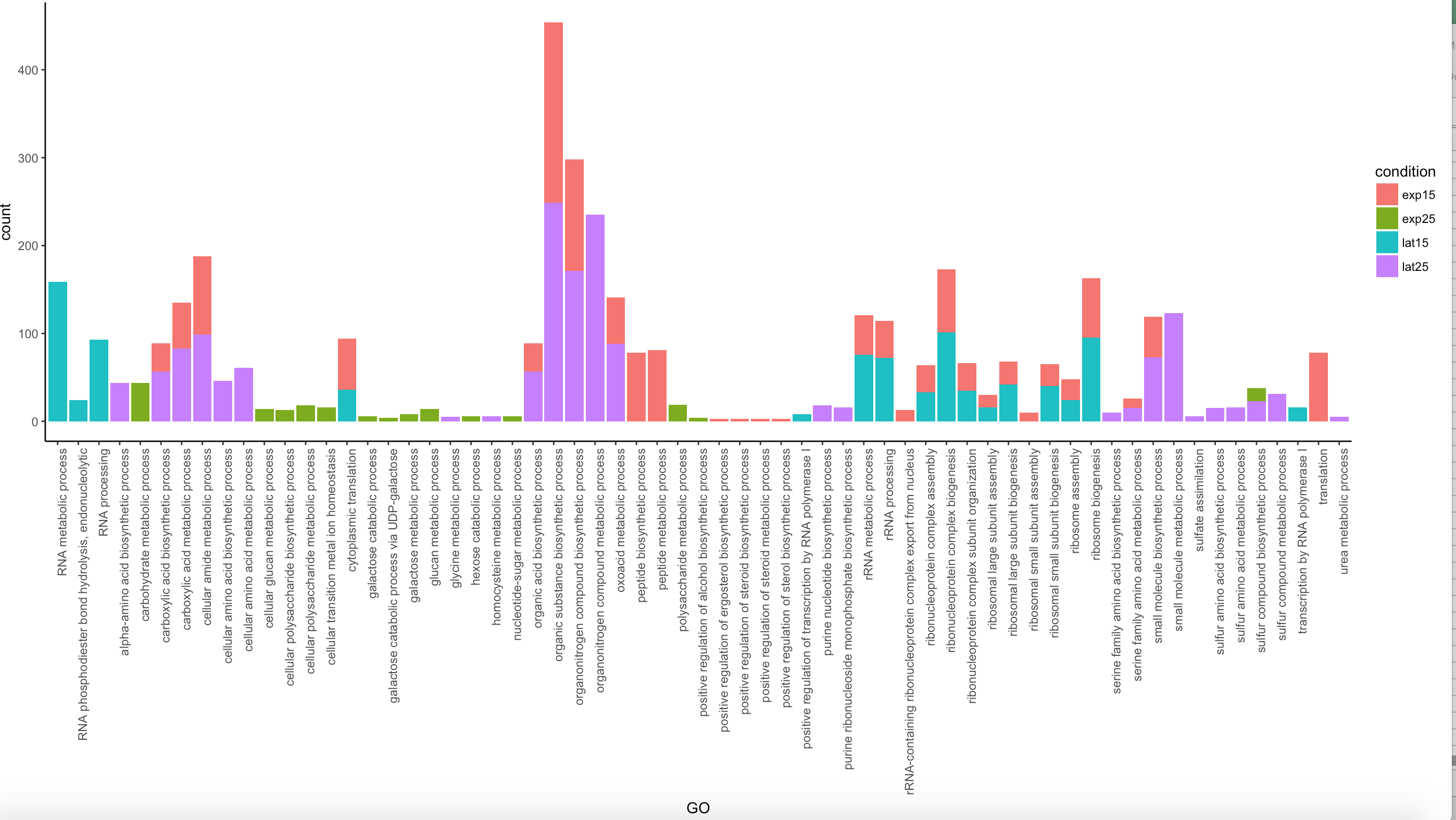

Supplement: FIGURE S2 — Overrepresented GO terms from the differentially expressed genes between H14A7 and AJ4. H14A7 global expression is enriched in these terms compared to AJ4. The number of genes that belong to each GO is represented in the bar in 4 different colors (red for overrepresentation in samples belonging to the exponential phase at 15°C, green for exponential at 25°C, blue for latency at 15°C, and violet for latency at 25°C). [file Image_2.TIFF]

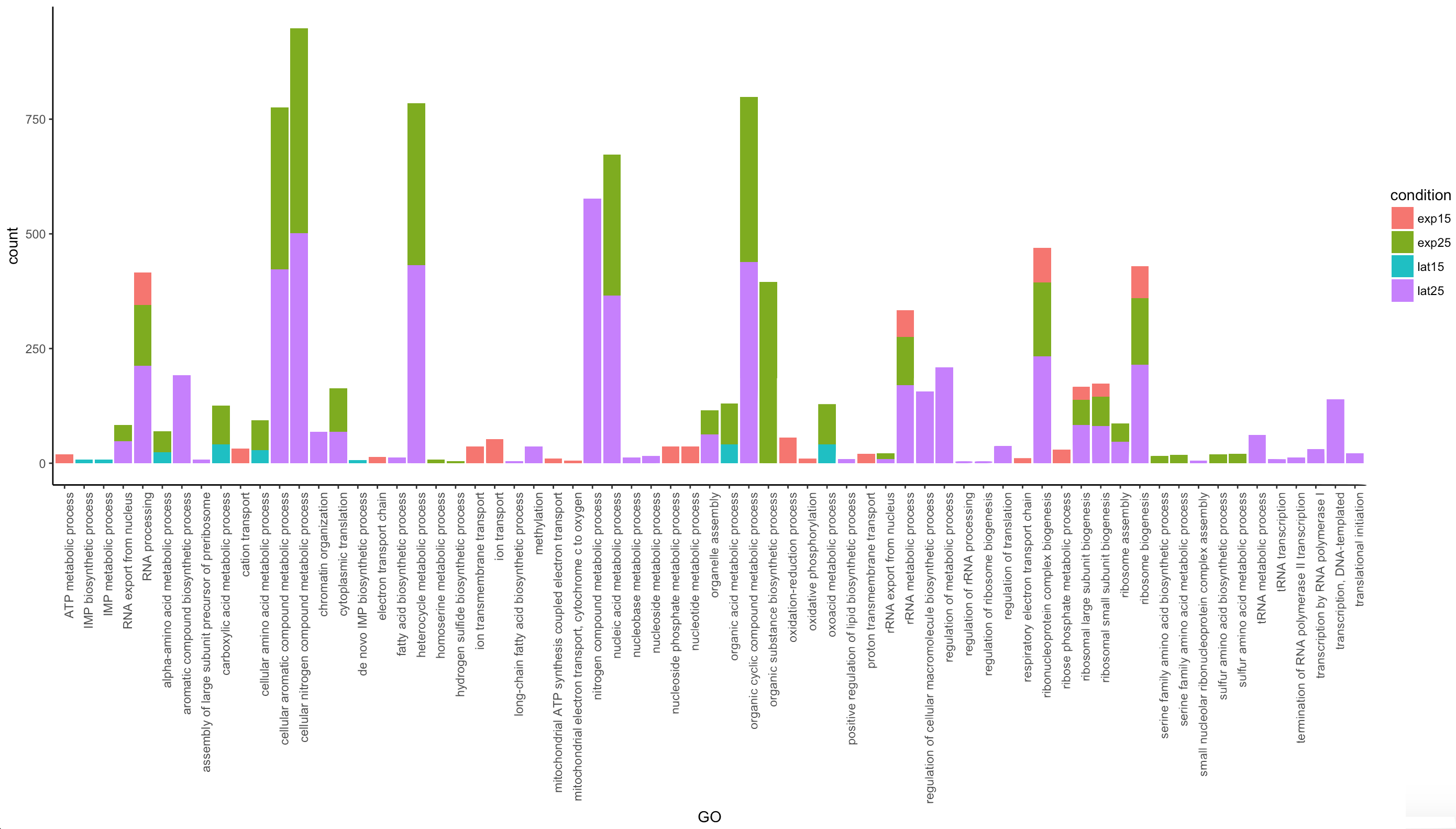

Supplement: FIGURE S3 — Overrepresented GO terms from the differentially expressed genes between AJ4 and H14A7. AJ4 global expression is enriched in these terms compared with H14A7. The number of genes that belong to each GO is represented in the bar in 4 different colors (red for overrepresentation in samples belonging to the exponential phase at 15°C, green for exponential at 25°C, blue for latency at 15°C, and violet for latency at 25°C). [file Image_3.TIFF]

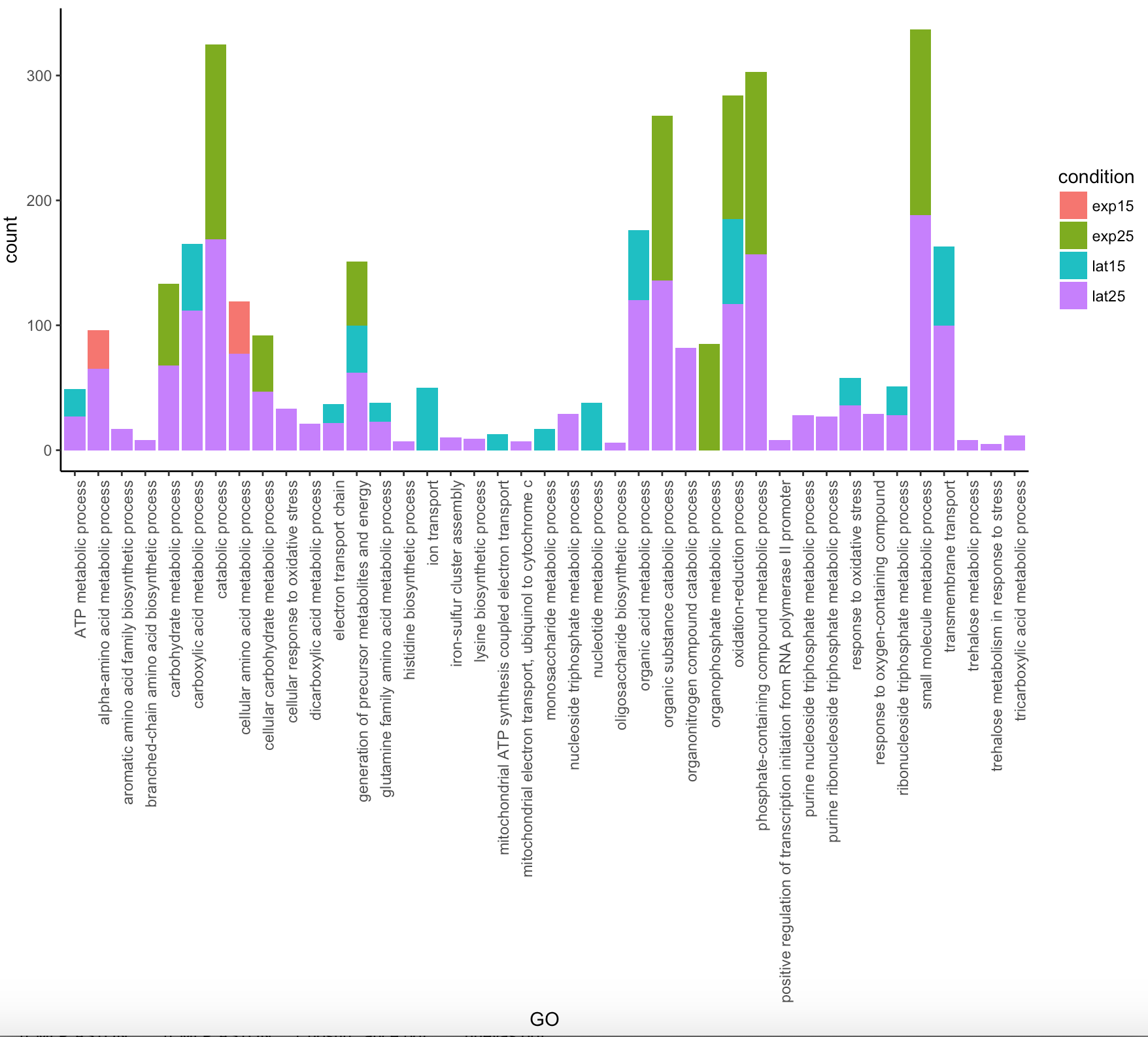

Supplement: FIGURE S4 — Overrepresented GO terms from the differentially expressed genes between H14A7 and BMV58. H14A7 global expression is enriched in these terms compared with BMV58. The number of genes that belong to each GO is represented in the bar in 4 different colors (red for overrepresentation in samples belonging to the exponential phase at 15°C, green for exponential at 25°C, blue for latency at 15°C and violet for latency at 25°C). [file Image_4.TIFF]

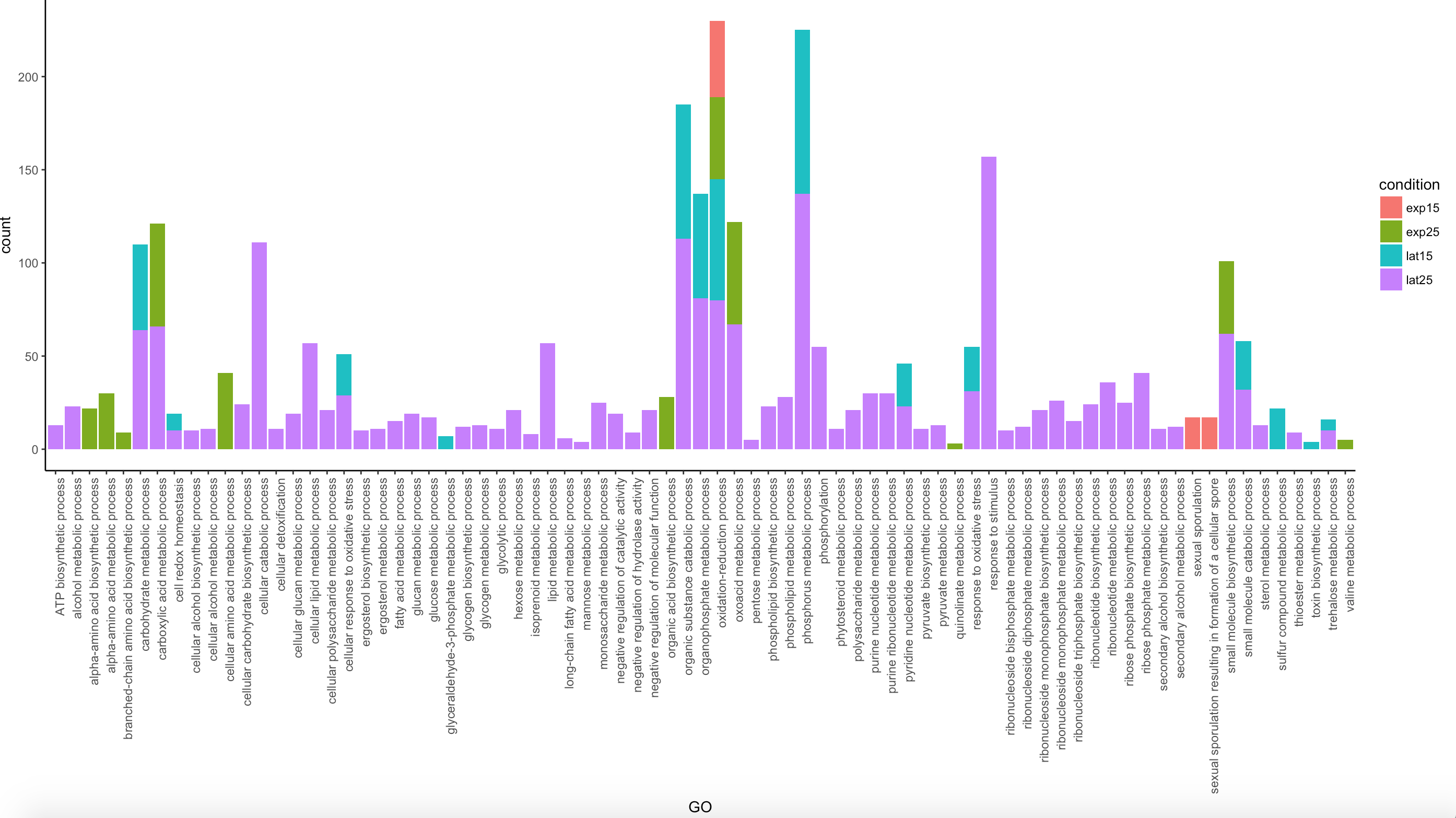

Supplement: FIGURE S5 — Overrepresented GO terms from the differentially expressed genes between BMV58 and H14A7. BMV58 global expression is enriched in these terms compared with H14A7. The number of genes that belong to each GO is represented in the bar in 4 different colors (red for overrepresentation in samples belonging to the exponential phase at 15°C, green for exponential at 25°C, blue for latency at 15°C and violet for latency at 25°C). [file Image_5.TIFF]
