# Supplementary material for: Differential Contribution of the Parental Genomes to a S. cerevisiae × S. uvarum Hybrid, Inferred by Phenomic, Genomic, and Transcriptomic Analyses, at Different Industrial Stress Conditions
Source: Front Bioeng Biotechnol. 2020 Mar 3;8:129. doi: 10.3389/fbioe.2020.00129 (PMC7062649; doi:10.3389/fbioe.2020.00129)
Supplement: TABLE S1 — PCR-RFLP analysis of 10 colonies isolates from the different hybrids obtained by sporulation. [file Table_1.pdf]

Table S1 PCR-RFLP analysis of 10 colonies isolates from the different hybrids obtained by sporulation

	APM3 **(II)**	**ITS (XII)**	***UGA3*** **(IV)**	***GSY1*** **(VI)**	***BRE5*** **(XIV)**
H3 D1	U	U	U	U	U
H3 D2	U	U	U	U	U
H3 D3	U	U	U	U	U
H3 D4	U	U	U	U	U
H3 F2	U	U	U	U	U
H3 F3	U	U	U	U	U
H3 G6	U	U	U	U	U
H3 G7	U	U	U	U	U
H3 G8	U	U	U	U	U
H3 G9	U	U	U	U	U
H4 A6	U	U	U	U	U
H4 A7	U	U	U	U	U
H4 A8	U	U	U	U	U
H4 A9	U	U	U	U	U
H4 I2	U	U	U	U	U
H4 I3	U	U	U	U	U
H4 I4	U	U	U	U	U
H4 I6	U	U	U	U	U
H4 I7	U	U	U	U	U
H4 I8	U	U	U	U	U
**H14 A6**	**CU**	**CU**			
**H14 A7**	**CU**	**CU**			
**H14 A8**	**C**	**C**	**C**	**CU**	
**H14 A9**	**C**	**CU**			
**H14 B1**	**C**	**C**	**C**	**CU**	
**H14 C1**	**C**	**CU**			
H14 D6	C	C	C	C	C
**H14 D7**	**C**	**C**	**C**	**CU**	
**H14 D8**	**CU**	**CU**			

Note: The isolates that remained being hybrids are marked in bold. U means that at this genomic region both alleles show *S. uvarum pattern*, C *S. cerevisiae* pattern and CU both species patterns.
