# Supplementary material for: Differential Contribution of the Parental Genomes to a S. cerevisiae × S. uvarum Hybrid, Inferred by Phenomic, Genomic, and Transcriptomic Analyses, at Different Industrial Stress Conditions
Source: Front Bioeng Biotechnol. 2020 Mar 3;8:129. doi: 10.3389/fbioe.2020.00129 (PMC7062649; doi:10.3389/fbioe.2020.00129)
Supplement: TABLE S2 — Total number of SNPs in AJ4, BMV58 and H14A7 strains. [file Table_2.pdf]

Supplementary Table S2. Total number of SNPs in AJ4, BMV58 and H14A7 strains

Strain	SNP type	Number	SNP/kb	SNPs %
AJ4	intergenic	4495		
AJ4	synonymous	1905		
AJ4	nonsynonymous	1952		
AJ4	total	8352		
**AJ4**	**SNP frequency**	**0,000673**	**0,673227**	**0,067%**
BMV58	intergenic	1894		
BMV58	synonymous	398		
BMV58	nonsynonymous	312		
BMV58	total	2604		
**BMV58**	**SNP frequency**	**0,000223**	**0,223386**	**0,022%**
H14A7	intergenic	7206		
H14A7	synonymous	2716		
H14A7	nonsynonymous	2274		
H14A7	total	12196		
**H14A7**	**SNP frequency**	**0,000507**	**0,506839**	**0,051%**
